# Environment‐Wide Association Study (E^n^WAS) of Prenatal and Perinatal Factors Associated With Autistic Traits: A Population‐Based Study

**DOI:** 10.1002/aur.2372

**Published:** 2020-08-23

**Authors:** Masoud Amiri, Sander Lamballais, Eloy Geenjaar, Laura M. E. Blanken, Hanan El Marroun, Henning Tiemeier, Tonya White

**Affiliations:** ^1^ Department of Child and Adolescent Psychiatry Erasmus MC University Medical Center Rotterdam Rotterdam The Netherlands; ^2^ Department of Epidemiology Erasmus MC University Medical Center Rotterdam Rotterdam The Netherlands; ^3^ The Generation R Study Group Erasmus MC University Medical Center Rotterdam Rotterdam The Netherlands; ^4^ Delft University of Technology Delft The Netherlands; ^5^ Department of Psychiatry Academic Medical Center Amsterdam The Netherlands; ^6^ Department of Psychology, Education & Child Studies, Erasmus School of Social and Behavioral Sciences Erasmus University Rotterdam Rotterdam The Netherlands; ^7^ Department of Social and Behavioral Sciences Harvard TH Chan School of Public Health Boston Massachusetts USA; ^8^ Department of Radiology and Nuclear Medicine Erasmus MC University Medical Center Rotterdam Rotterdam The Netherlands

**Keywords:** autism spectrum disorder, autistic traits, environment‐wide‐association study, exposure, perinatal, prenatal

## Abstract

A combination of genetic and environmental factors contributes to the origins of autism spectrum disorder (ASD). While a number of studies have described specific environmental factors associating with emerging ASD, studies that compare and contrast multiple environmental factors in the same study are lacking. Thus, the goal of this study was to perform a prospective, data‐driven environmental‐wide association study of pre‐ and perinatal factors associated with the later development of autistic symptoms in childhood. The participants included 3891 6‐year‐old children from a birth cohort with pre‐ and perinatal data. Autistic symptoms were measured using the Social Responsiveness Scale in all children. Prior to any analyses, the sample was randomly split into a discovery set (2920) and a test set (921). Multiple linear regression analyses were performed for each of 920 variables, correcting for six of the most common covariates in epidemiological studies. We found 111 different pre‐ and perinatal factors associated with autistic traits during childhood. In secondary analyses where we controlled for parental psychopathology, 23 variables in the domains of family and interpersonal relationships were associated with the development of autistic symptoms during childhood. In conclusion, a data‐driven approach was used to identify a number of pre‐ and perinatal risk factors associating with higher childhood autistic symptoms. These factors include measures of parental psychopathology and family and interpersonal relationships. These measures could potentially be used for the early identification of those at increased risk to develop ASD.

**Lay Summary:**

A combination of genetic and environmental factors contributes to the development of autism spectrum disorder (ASD). Each environmental factor may affect the risk of ASD. In a study on 6‐year‐old children, a number of pre‐ and perinatal risk factors were identified that are associated with autistic symptoms in childhood. These factors include measures of parental psychopathology and family and interpersonal relationships. These variables could potentially serve as markers to identify those at increased risk to develop ASD or autistic symptoms. ***Autism Res** 2020, 13: 1582–1600*. © 2020 International Society for Autism Research, Wiley Periodicals, Inc.

## Introduction

Autism spectrum disorder (ASD) is a neurodevelopmental disorder with an onset typically within the first 2 years of life [Barger, Campbell, & McDonough, 2013] and is characterized by abnormalities in social behavior, communication and repetitive and stereotypic behavior [Barger et al., 2013]. The heritability of ASD is relatively high, with estimates ranging from 64% to 91% [Tick, Bolton, Happe, Rutter, & Rijsdijk, 2016]. While genetics play a substantial role in the etiology of ASD, a number of early environmental risk factors have also been identified that may contribute to the pathogenesis [Newschaffer, Fallin, & Lee, 2002]. Environmental variables associated with ASD or autistic symptoms include maternal and paternal age [Sandin et al., 2012], prenatal maternal vitamin D deficiency [Sotodehasl, Tamadon, & Malek, 2018; Vinkhuyzen et al., 2017], folate deficiency [Krsicka et al., 2017], prenatal maternal selective serotonin reuptake inhibitor use during pregnancy [El Marroun et al., 2014], and the ethnic background of the family [Becerra et al., 2014], although ethnic differences in interpreting autism symptom checklists could confound the results of the latter [Cheon et al., 2016; Hus, Bishop, Gotham, Huerta, & Lord, 2013; Moul, Cauchi, Hawes, Brennan, & Dadds, 2014].

The incidence of ASD has risen over the last few decades [Jensen, Steinhausen, & Lauritsen, 2014], which can partially be explained by better mental health care coverage, less stigma, greater awareness, and altered diagnostic criteria [Rutter, 2005]. However, the rise could also be partially related to parallel changes in environmental factors, such as the trend of increasing parental age at child birth [Parner et al., 2012] and the viability of prematurely born infants [Limperopoulos et al., 2008]. This emphasizes the need to further elucidate early environmental risk factors for emerging ASD. Since autism‐related symptoms have previously been suggested to lie on a continuum in the population [Constantino & Todd, 2003], the Social Responsiveness Scale (SRS) can be used to assess autistic traits across the spectrum within the general pediatric population [Moul et al., 2014].

While, genome‐wide association studies (GWAS) have helped in the understanding of the genetic components of complex traits and the identification of different loci that may be associated with diseases and clinical symptoms [Hindorff et al., 2009]. GWAS have revealed thousands of single nucleotide polymorphisms (SNPs) associated with many diseases; however, many questions remain regarding the heritability and potential mechanisms that result in complex and common diseases [Hindorff et al., 2009; Maher, 2008]. Environmental exposures may also have a major impact on molecular and cellular systems for many diseases [Maher, 2008]. Environment‐wide association studies (E^n^WAS) could provide a practical method to test a variety of exposures in human environment in a unbiased manner, similar to GWAS tests for genetic effects [Patel, Bhattacharya, & Butte, 2010]. Examples of E^n^WAS applications have been shown for Type 2 diabetes [Patel et al., 2010], metabolic syndrome [Lind, Riserus, Salihovic, Bavel, & Lind, 2013], peripheral arterial disease [Zhuang et al., 2018] and blood pressure [McGinnis, Brownstein, & Patel, 2016].

Although a broad range of environmental risk factors have been found to be associated with ASD, their individual effects tend to be small, typically with odds ratios less than two [Karimi, Kamali, Mousavi, & Karahmadi, 2017]. Most previous epidemiological studies have focused on a single or several exposures per hypothesis [Arora et al., 2017; Mezzacappa et al., 2017; Morales‐Suarez‐Varela, Peraita‐Costa, & Llopis‐Gonzalez, 2017]; however, prospective cohort studies provide the opportunity to evaluate a broader range of exposures and factors. Thus, it was the goal of this study to evaluate the risk conveyed by a plurality of factors on autistic traits using a data‐wide approach.

We analyzed data that were prospectively collected during pre‐ and perinatal life to determine early factors associated with the later development of autistic traits. We applied a data driven approach with the use of a “discovery” and “test” set to reduce bias and false positives and to add confidence that the observed differences in the original study are true within the context of the study population [Hernandez Cordero et al., 2018; Holzinger et al., 2017; Kraft, Zeggini, & Ioannidis, 2009; Shen et al., 2017]. We divided our sample a priori into a discovery set and test set with 75% and 25% of the participants, respectively.

## Methods

### *Participants*

**Figure 1:**
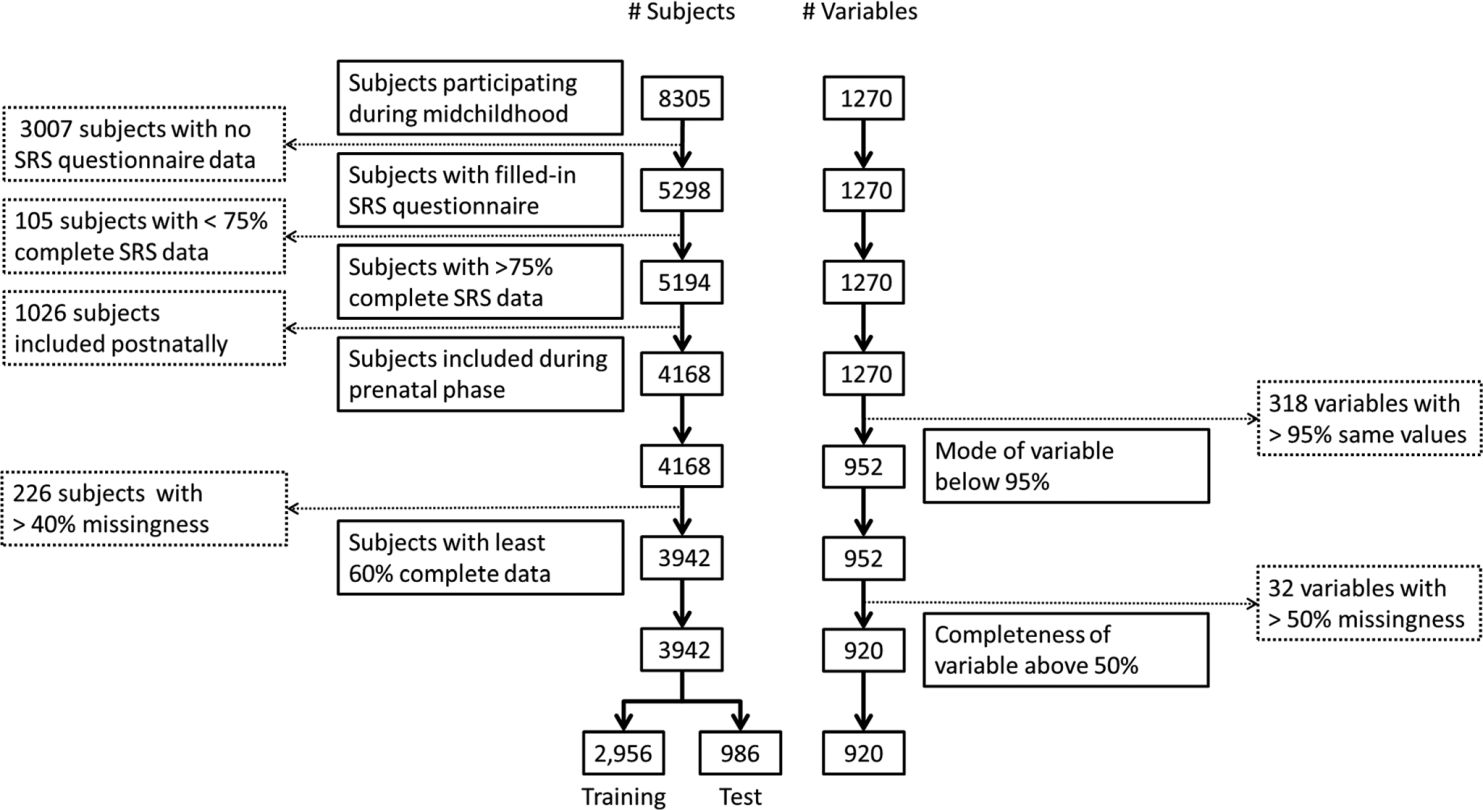
Flow chart of the inclusion and exclusion of the study population.

The E^n^WAS was performed within the Generation R Study, a prospective cohort study based in Rotterdam, the Netherlands [Kruithof et al., 2014; Tiemeier et al., 2012]. The Generation R Study is a large, prospective population‐based birth‐cohort in which all pregnant women who were living within a well‐defined region in Rotterdam (defined by postal codes) with a delivery data between April 2002 and January 2006 were invited to participate. Parents and their children participated in a wide range of measures as described below. Out of 9745 children born, 8305 participated in the visit at the age of 6 [Jaddoe et al., 2012]. Of these, the mothers of 5194 children completed the SRS, a questionnaire that assesses autistic symptoms [Constantino et al., 2003]. After removal of participants with postnatal inclusion or surpassing the threshold of missing data, the final sample consisted of 3942 children (Fig. 1). Ethical approval was obtained from the Erasmus Medical Center Medical Ethics Committee, and written informed consent was obtained from the primary caregivers.

Examinations were performed at each visit during early pregnancy, mid pregnancy and late pregnancy and included height (only assessed at the first visit), weight and blood pressure measurements of both parents. In addition, the mothers received four questionnaires during pregnancy. In early pregnancy, mothers reported on medical and family history, previous pregnancies, quality of life, lifestyle habits, housing conditions, ethnicity, and educational level. In midpregnancy, mothers received a specific food frequency questionnaire and reported on diet, including macronutrients and micronutrients. In midpregnancy mothers also reported on current pregnancy, quality of life, lifestyle habits, and psychopathology. And in late pregnancy, mothers reported on factors associated with her current pregnancy, quality of life, lifestyle habits, working conditions, household income, and self‐esteem. Partners received one questionnaire during pregnancy and reported on their medical history, family history, lifestyle habits, educational level, and psychopathology). Blood samples of mothers and partners were collected in early pregnancy and cord blood was collected at birth [Jaddoe et al., 2010].

### *Measurement of Autistic Traits*

The SRS is a questionnaire that measures autistic traits in children between 4 and 18 years of age [Constantino & Gruber, 2005; Constantino, Przybeck, Friesen, & Todd, 2000]. The SRS was completed by the parents when the children were approximately 6 years of age. Each item in the questionnaire is scored from 0 (“never true”) to 3 (“almost always true”). Higher scores indicate more autistic symptoms. The standard SRS has 65 items; however, due to time constraints, an 18‐item version of the SRS was used in the Generation R Study. The SRS was excluded if over 25% of the questions were missing; otherwise a weighted total score was calculated based on the number of nonmissing items. The 18‐item version has been shown to correlate highly with the full SRS version [Roman et al., 2013]. For example, we evaluated a sample of 3857 children aged 4–18 who took part in the Social Spectrum Study in the Netherlands, the correlation between total scores derived from the 18 item SRS short‐form and the complete SRS was 0.95 [Blanken et al., 2015]. The correlation between total scores derived by the SRS short‐form and the complete SRS in the Missouri Twin Study [Constantino & Todd, 2003] was 0.93 in monozygotic male twins and 0.94 in dizygotic male twins. In a sample of 2719 children from the Interactive Autism Network's [Daniels et al., 2012] the corresponding correlation was 0.99.

### *ASD Diagnoses*

General practitioners serve as the source for the central medical records in The Netherlands, including information on treatment by medical specialists. A diagnosis of ASD was based on the clinical consensus by a specialized multidisciplinary team and is reported by the family physician. To confirm a diagnosis of ASD, we first screened children based on three sources of information; (1) a history of ASD in the child provided by the parents, and( 2) scoring above the clinical cutoff on the SRS, (3) children who scored in the top 15% of the child behavior checklist (CBCL) underwent additional screening using the Social Communication Questionnaire (SCQ). For those children who screened positive (*n* = 186) we obtained medical records to confirm the diagnosis of ASD (*n* = 84) (T. White et al., 2018].

### *Environmental‐Wide Variables*

Data that was available from the prenatal and perinatal measurements within the Generation R Study were used. The majority of the data consisted of questionnaires that were sent to the mothers during pregnancy. To increase the interpretability of the data, we organized the variables into topical domains. These domains were defined by the authors and were used to improve interpretation of the dataset and results. The domains were constructed to capture distinct groups of variables. The following domains were specified in this study: (1) parental health, (2) parental psychopathology, (3) sociodemographic and migration factors of parents and grandparents, (4) parental prenatal lifestyle and life events, (5) parental exposures to nutrition, toxins and other chemicals, (6) family and rearing, (7) maternal expectations regarding the child, (8) maternal biomarkers from prenatal serum, (9) perinatal complications and obstetrics, and (10) cord blood biomarkers. While the variables in these domains cover a broad spectrum, ranging from maternal and child biomarkers to parental behaviors, some variables were only measured once (i.e., serum levels of folate, vitamin D, fatty acids) and other variables had very low frequency (use of cocaine, opioids, and other hard drugs).

### *Parental Health*

At each visit during pregnancy, maternal weight, height and systolic and diastolic blood pressure [Silva et al., 2008]. Prepregnancy contraceptive use, that is, use of condoms, contraceptive pills, and intrauterine devices were assessed in the first maternal pregnancy questionnaire. The first maternal questionnaire also contained the Short Form Survey‐12 [Ware Jr., Kosinski, & Keller, 1996], which assesses problems with mobility, daily activities, exercise and pain. It also assessed the presence of infections, numerous inflammatory conditions and gynecology‐related problems such as bleeding after intercourse, during the 3 months before filling in the questionnaire. The parents were also asked about whether they had ever been diagnosed with any medical conditions such as asthma, hypertension, and thrombosis. Furthermore, we also included the questions of the somatization scale of the Brief Symptom Inventory (BSI) [Derogatis & Melisaratos, 1983], a 53‐item self‐report questionnaire that assesses psychological distress and was filled in by both the mothers and the fathers.

### *Parental Psychopathology*

The bulk of this domain consisted of maternal and paternal data on BSI questions and subscales: obsessive–compulsive, interpersonal sensitivity, depression, anxiety, hostility, phobic anxiety, paranoid ideation, and psychoticism [Derogatis & Melisaratos, 1983]. This domain also included the Global Severity Index (GSI), the weighted sum score of all BSI items. The questionnaires also contained separate vignettes on diagnoses related to depression (including manic episodes), anxiety disorders, psychosis, eating disorders, and addiction [Micali et al., 2012]. Finally, a modified version of the Rosenberg's Self‐Esteem scale was administered during the third trimester. This instrument consisted of questions such as “in most areas my life is ideal” and “I sometimes think I am worthless,” judged on a 5‐point Likert scale.

### *Sociodemographic and Migration Factors of Parents and Grandparents*

National origin of the parents and grandparents assessed by country of birth [Jaddoe et al., 2006]. The following non‐Dutch ethnic groups were categorized: Moroccan, Turkish, Cape Verdean, Antillean and Surinamese. Participants of other ethnic backgrounds were either classified as “Other western” or “Other nonwestern” [Troe et al., 2008]. Ethnicity of the child was determined through the following algorithm: (1) if both parents were born in the Netherlands, then the ethnicity of the child was considered Dutch; (2) if one of parents was born in a country other than the Netherlands, then ethnicity of the child was selected by the country of birth of that parent; or (3) if both parents were born in two different countries other than the Netherlands, then the country of the mother was selected for the ethnicity of the child.

A series of questions related to ethnic and social identity, inquiring to what extent respondents felt Dutch or part of the Dutch culture, what ethnicity they felt they identify with, how much time they spent with ethnically Dutch people, and whether they felt treated fairly by society. These questions were included due to the abundance of different ethnicities and immigrants that live in the city of Rotterdam. We also assessed whether the parents or the grandparents were born outside of The Netherlands, and whether they moved to The Netherlands before or after the age of 15 years old.

Parental education was assessed through questionnaires and scaled down to three levels (low, intermediate, and high). The same was done for educational level of the grandparents from the maternal side. Furthermore, during pregnancy we assessed marital status, household income (scaled down to three levels), and parental employment. In addition, we asked about employment of the grandparents and whether the grandparents were divorced during the mother's childhood.

Three questions related to religion were asked: whether the respondent was brought up in a specific religion, whether they practice a religion now or belong to a religious community, and how often they attend a religious meeting. Finally, several questions were included on the characteristics of the neighborhood the participants live in, for example, the presence of vandalism and graffiti.

### *Parental Prenatal Lifestyle and Life Events*

The domain of prenatal lifestyle and life events contained variables describing the home environment and parental lifestyles before the birth of the child. This included questions about pets, parental smoking, parental alcohol use, parental drug use, maternal coffee consumption, maternal sexual contacts and who resided in the home during pregnancy and at the time of the delivery. An 18‐item questionnaire on delinquent behavior was also filled in by both mothers and fathers [van der Laan & Blom, 2006]. Mothers also filled in the List of Threatening Events [Rosmalen, Bos, & de Jonge, 2012], a modified and translated version of the Social Readjustment Rating Scale [Holmes & Rahe, 1967] that evaluates the extent of impactful life events such as the loss of a child. Finally, Long Lasting Difficulties Inventory was used to assess presence of long‐term stressors [van Eck, Berkhof, Nicolson, & Sulon, 1996].

### *Parental Exposures to Nutrition, Toxins, and Other Chemicals*

The mothers completed a modified version of the validated semiquantitative food frequency questionnaire (FFQ) of Kipstein‐Grobusch and colleagues [Klipstein‐Grobusch et al., 1998]. Due to the length of the FFQ we only included information on single food categories and nutrients. Main food categories were: (1) potatoes and other tubers, (2) vegetables, (3) legumes, (4) fruits, (5) dairy products, (6) cereals and cereal products, (7) meat and meat products, (8) fish and shellfish, (9) eggs and egg products, (10) fat, (11) sugar and confectionery. (12) cakes, (13) nonalcoholic beverages, (14) condiments and sauces, and (15) soups and bouillon. Information on nutrients was calculated based on the Dutch Food‐Composition Table 2006 [Netherlands‐Nutrition‐Centre, 2006], as described elsewhere [Heppe et al., 2013]. These included measures such as the total daily caloric intake, including the amounts of carbohydrates, saturated fats, specific minerals, vitamins, and additional measures [Neelakantan et al., 2016; Steenweg‐de Graaff et al., 2014].

Information on self‐reported prepregnancy vitamin supplementation, thyroid medication and folic acid supplementation was collected in early pregnancy. Both mothers and partners answered several questions related to occupational usage of substances like paint and heavy metals. Finally, measurements of the exposure to air pollutants, including measures of NO_2_, NO_x_, PM10 and PM25, were calculated based on the reported household location [Guxens et al., 2016], which have been described elsewhere [Eeftens et al., 2012]. In brief, air pollution was monitored between October 2008 and January 2011 and mapped with land‐use regression models. Consequently, estimates were back‐extrapolated based on annual average air pollution concentrations to estimate the pregnancy‐average concentrations.

### *Family and Rearing*

During the third trimester of pregnancy, questionnaires on current family functioning and maternal childhood upbringing were collected. The majority of variables within the family domain contained questions from the Family Assessment Device (FAD) [Epstein, Baldwin, & Bishop, 1983] and the Egna Minnen Beträffende Uppfostran (EMBU) questionnaire [Ross, Campbell, & Clayer, 1982]. The FAD assesses family functioning. Both mothers and partners completed the FAD of which one computes the General Functioning scale. The EMBU is specifically aimed at childhood rearing in mothers, with questions such as “my father tried to encourage me to be the best.” Separate questions were used to describe parenting of the mother and father. Finally, the Childhood Trauma Questionnaire was administered, containing questions such as “I thought my parents wished I had never been born” and “I got hit or beaten so badly that it was noticed by someone like a teacher, neighbor, or doctor” [Bernstein et al., 1994].

### *Maternal Expectations Regarding the Child*

The domain on maternal expectations contained questions from two questionnaires: the Pregnancy Outcome Questionnaire [Theut, Pedersen, Zaslow, & Rabinovich, 1988] and the Cohler's maternal attitude scale [Cohler, Weiss, & Grunebaum, 1970]. The Pregnancy Outcome Questionnaire assessed pregnancy‐specific anxiety and was part of the first pregnancy questionnaire. The maternal attitude scale focuses on child care attitude and describes how the mother envisioned having a child and how she expected her child to behave. For example, questions about the baby's emotions and crying were included. This questionnaire was administered during the third trimester of pregnancy.

### *Maternal Biomarkers*

Each biomarker that was used in this study has been cleaned and most have been used in other published epidemiologic studies, although only a few in studies of ASD. The biomarkers extracted from blood were available for nearly all mothers (97%) [Jaddoe et al., 2010]. However, biomarkers extracted from urine were collected only between February 2003 and November 2005 and were only available for 4000 mothers (three biomarkers). All biomarkers have been assessed for quality [Kruithof et al., 2014]. The maternal biomarkers and the associated references include: antitissue transglutaminase antibody concentrations [Jansen et al., 2014], thyroid peroxidase antibodies [Korevaar et al., 2013], C‐reactive protein [de Jonge et al., 2011], folic acid [Ars et al., 2016], homocysteine [Bergen et al., 2012], plasminogen activator inhibitor‐2 [Bouwland‐Both et al., 2013], placental growth factor [Korevaar et al., 2014], thyroid stimulating hormone and other thyroid hormones [Korevaar et al., 2016], active vitamin B12 [Ars et al., 2016], and fatty acids [Steenweg‐de Graaff et al., 2016]. From the available urinary biomarkers, iodine [Ghassabian et al., 2014], creatinine, and tetrahydrocannabinol were included in the study [El Marroun et al., 2016].

### *Perinatal Complications and Obstetrics*

The perinatal and obstetrics domain contained several obstetric‐related measures, such as the type of delivery, gestational age at birth, parity, method of conception, location of delivery, use of sedation, APGAR scores at 1 and 5 min, blood loss during the first and second half of the pregnancy, meconium in amniotic fluid, premature rupture of membranes, intrauterine growth restriction, breech presentation, preeclampsia, gestational diabetes, fetal stress, and twin birth. This information was retrieved from records of hospitals and midwife practices [Coolman et al., 2010].

### *Cord Blood Biomarkers*

At birth, 30 ml of cord blood was collected for 67% of the children. The cord blood was tested for a wide range of biomarkers, including thyroid stimulating hormone and other thyroid hormones [Medici et al., 2012], C‐reactive protein [Sonnenschein‐van der Voort et al., 2013], folate [Krsicka et al., 2017], homocysteine [van der Valk et al., 2013], placental growth factor [Bautista Nino et al., 2015], soluble fms‐like tyrosine kinase‐1 [Bautista Nino et al., 2015], and total and active vitamin B12 [van der Valk et al., 2013].

## Data Preparation

We opted to include both individual items and composite scores from scales. This was done as individual items could associate with the SRS score different than the underlying construct that a composite score would measure. Further, since we performed iterative multiple linear regression with each variable separately, the only penalty to this approach was the additional tests, which we corrected for and thus applied conservative multiple testing correction. To prepare the data for analysis, we undertook several steps on a per‐variable basis across the entire dataset. At a per‐variable level we (1) recoded conditional questions, (2) classified each variable as unordered categorical, ordered categorical or continuous, (3) split questions with a “Do not know” option, and (4) reduced the number of categories for categorical variables if they contained little information. At the dataset level we (1) excluded items with little information, (2) removed participants with more than 60% missing data, and (3) removed variables with more than 50% missingness. Each of these steps is described below.

First, conditional questions are questions that were asked based on the participant's answer on a previous question. For example, the question “Do you still smoke?” was only asked if the participant answered “Yes” to the previous question: “Have you ever smoked?” Conditional questions only have data for the subset of participants who were asked the question. We therefore merged conditional questions with their parent questions to capture a wider range of variance. For example, the new smoking variable becomes: “Never smoked,” “Past smoker,” “Current smoker.”

Second, each variable in the dataset was assigned the label of unordered categorical (e.g., ethnicity), ordered categorical (e.g., education level) or continuous (e.g., birth weight). Third, a number of questions, such as the question “What was your birth weight?” to the mother, had an option for “Do not know.” We reasoned that this answer does not contain information, so we recoded these as missing. Finally, categorical items tended to have sparsely populated categories, which would undermine the statistical analyses. We therefore inspected every categorical variable and merged categories along a fixed algorithm. For example, variables with 4 or more options where most answers leaned to one side (e.g., from “Never” to “Always”) were recoded along the lines of “Never,” “Between never and frequent” and “Frequent.” Variables with 5 or more options where most answers were in the center (e.g., from “Strongly disagree” to “Strongly agree”) were recoded to “Left of middle”, “Middle,” “Right of middle.”

A number of measures were collected only in subsets of the whole population, and not all women enrolled during the first trimester of pregnancy and thus were unable to partake in all available measures. Furthermore, a number of questionnaire items had low variance in the data, such, as questions on whether the child has specific rare diseases. To prepare the dataset, we undertook a number of cleaning steps (Fig. 1). First, in order to avoid finding rare, inflated effects we removed all variables where more than 95% of all respondents had the same answer. Second, to ensure that participants had sufficient available information we removed subjects who had more than 60% missingness on all remaining variables. Finally, we further excluded variables that still had over 50% missing data. We varied these thresholds and did not find statistically significant influences on the results, and the specified thresholds were chosen to maximize the number of participants in the sets.

### *Statistical Analysis*

We initially performed a nonresponse analysis to compare the 3942 participants in the final sample to the 4363 participants that were excluded from analysis. The groups were compared on maternal age at birth, child ethnicity, sex of the child, and SRS score for those with SRS score data available.

Due to the breadth of measures used, the data showed structural patterns of missingness that prevented the inclusion of all data into a single model. Instead, we constructed a separate linear regression model for each of the 920 variables in the dataset with the mean SRS item score as the outcome. The SRS score distribution was skewed, thus to improve model fitting we applied a square root transformation to the distribution (see Fig. S1). The base model also included the age at which the SRS data was collected. In addition, we split the sample into a discovery set and a test set. The general flow of the analyses were as follows. First, all regression models were tested in the discovery set, which consisted of 75% of the total sample. Multiple testing was accounted for using the false discovery rate (FDR). Variables that passed the FDR threshold were further analyzed in the test set (the remaining 25% of the total sample), and we report the statistically significant variables that survived FDR correction for both the discovery and test set. We also present results using the more conservative Bonferroni correction for multiple testing. The rationale for the use of a discovery and test set was to increase the external validity of the findings.

Although each variable was tested in a separate model, we still aimed to account for residual confounding. We reasoned that extensive epidemiological research on antecedents of ASD exist, and so our goal was to find the most common covariates in epidemiological studies of ASD in the literature. These covariates were determined by performing a PubMed search with the term “odds ratio autism” and by recording and selecting the most common covariates. Within the PubMed search results for “odds ratio autism” up to December 2015, 73 epidemiological studies on ASD were identified out of 325 research results (Table S1). Thus, we created a second set of regression models that were corrected for the following covariates: age at which the SRS data was collected, maternal age at birth, maternal education, maternal ethnicity, child sex, parity of the pregnancy, and birth year. Paternal age was also a commonly used covariate in literature, but we did not include it due to its strong correlation with maternal age. We performed additional analyses in which included birth weight and gestational age, as these variables were also commonly used as covariates in the literature, although they also could be considered mediators in the pathway. We performed linear regression analyses to show the relationship between autistic symptoms and the identified covariates.

Further sensitivity analyses were performed to address the consistency of the associations. First, based on the literature we reasoned that parental psychopathology was likely a predictor of the SRS score. We thus created an additional set of regression models that were corrected for the maternal BSI sum score as obtained from the first questionnaire administered during pregnancy. Items that were within the domains parental health and parental psychopathology were excluded from these sensitivity analyses. Second, we identified that migrant status could be an important role in the main findings, which likely reflects differences in how the mothers completed the SRS. Thus, we reanalyzed all data in only the children with two ethnically Dutch parents. Due to the much lower number of participants in this set, we combined the discovery and test sets. Furthermore, we only performed this analysis with the goal of assessing which variables identified in the main analysis would remain statistically significant.

All analyses were performed in R (version 3.2.3) [R Development Core Team, 2016]. We imputed the missing values of all covariates using chained equations with the mice package in R [van Buuren & Groothuis‐Oudshoorn, 2011].

## Results

### *Study Population*

A flow chart of the inclusion for the study population is shown in Figure 1 and demographic and behavioral characteristics of the population are shown in Table 1. Participants that had been excluded from the final sample due to missing data differed from the children that were included in the analysis. The excluded children were more likely to have higher SRS scores (*p* < 0.0001, mean difference = 0.05 points) and to be born from younger mothers (*p* < 0.0001, mean difference = 2.1 years), and to have mothers of nonwestern descent (*p* < 0.0001, 47.1% vs. 22.7%). There was no statistically significant difference in the proportion of sexes (*p* = 0.11).

**Table 1:** Characteristics of the Discovery and Test Sets

	Discovery set			Test set		
Characteristics	*N*	%	Mean (*SD*)	*N*	%	Mean (*SD*)
Age in years at SRS			6.10 (0.43)			6.12 (0.42)
Score per SRS item			0.22 (0.23)			0.23 (0.26)
Boys	1450	49.1		502	50.9	
Ethnicity child						
Dutch	2034	68.9		652	66.1	
Other western	267	9.0		95	9.6	
Nonwestern	653	22.1		239	24.2	
Gestational age			39.93 (1.70)			39.85 (1.88)
Birth weight (g)			3447 (559)			3441 (572)
Cohort						
2002	273	9.2		93	9.4	
2003	837	28.3		283	28.7	
2004	961	32.5		309	31.3	
2005	871	29.5		297	30.1	
2006	14	0.5		4	0.4	
Maternal education				971		
Lower	134	4.6		58	6.0	
Middle	1040	35.9		346	35.6	
Higher	1722	59.5		567	58.4	
Maternal age			31.36 (4.46)			31.54 (4.42)
Parity number						
0	1801	61.0		594	60.4	
1	867	29.4		276	28.0	
2+	284	9.6		114	11.6	

SRS: Social Responsiveness Scale.

### *Univariate Associations With SRS*

**Figure 2:**
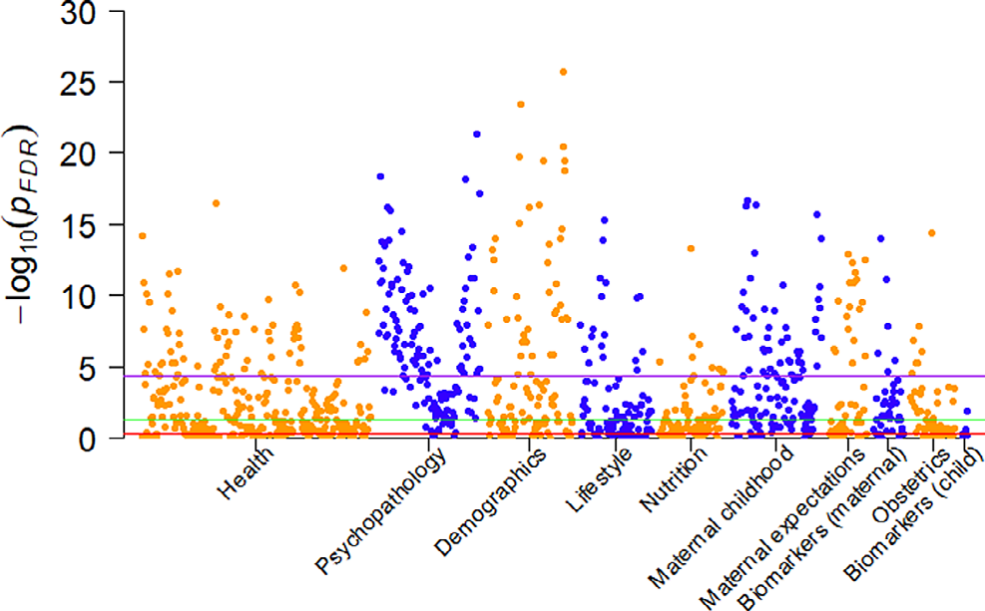
Manhattan plot of false discovery rate (FDR)‐corrected minus log 10 *p*‐values of the iterative regression analysis. Each group of colored dots represents a domain. The green horizontal line marks the 0.05 uncorrected threshold. The red horizontal line marks the 0.05 FDR‐corrected threshold. The purple horizontal line marks the 0.05 Bonferroni‐corrected threshold.

**Figure 3:**
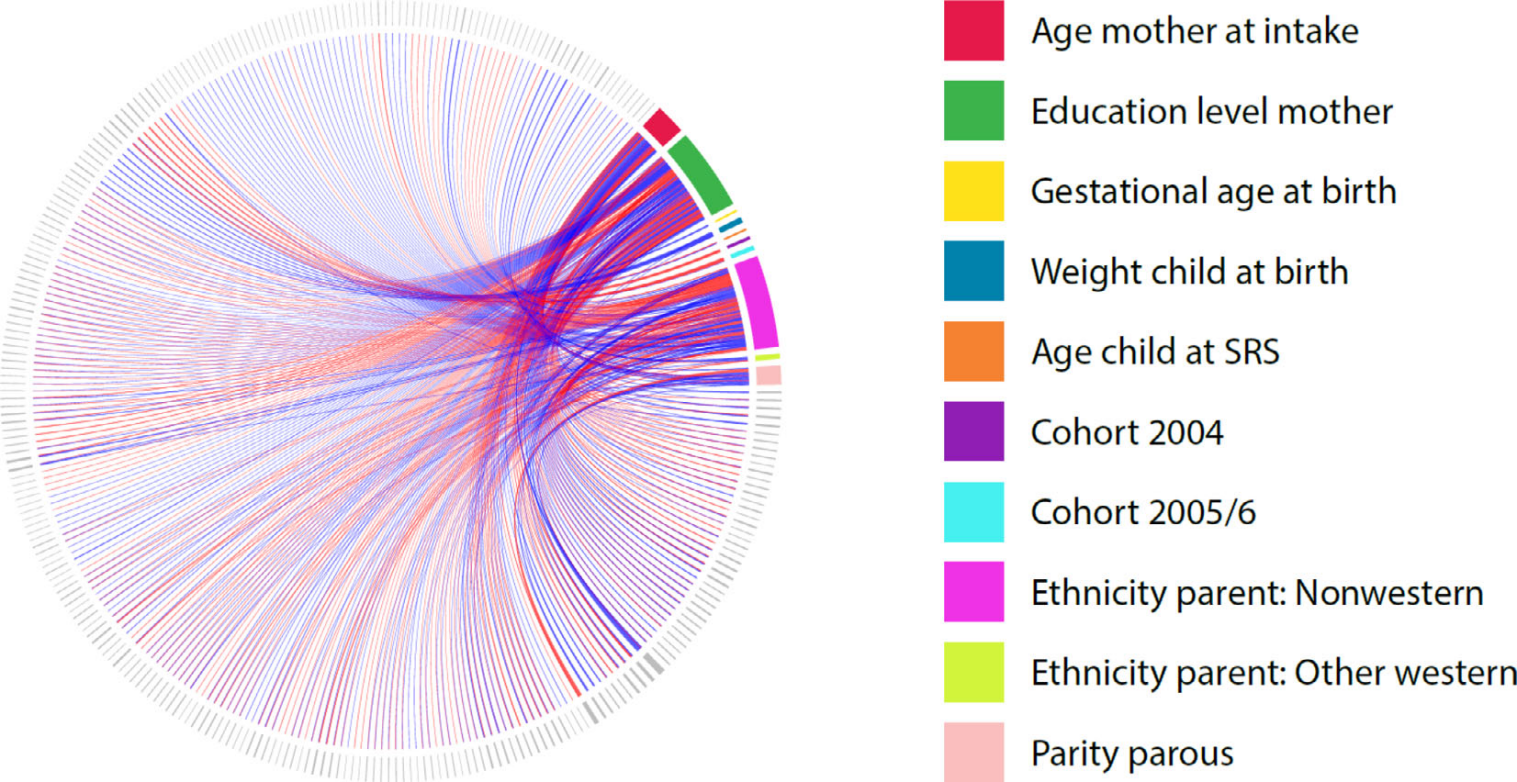
Plot demonstrating the high covariance structure between the different variables used in the analyses. As there were too many variables to list all in the figure, the purpose is to show the high correlation between multiple variables in the E^n^WAS. Red indicates a positive correlation whereas blue indicates a negative correlation. The legend reflects the different covariates used in the study.

The distribution of the square‐root transformed SRS is shown in Figure S1. Correcting only for the age of the child when the SRS was administered, we iteratively evaluated the associations between the SRS score and the data‐wide variables (Fig. 2). In total, 580 out of 917 variables remained statistically significant after FDR correction in the discovery cohort. The high proportion of statistically significant findings suggested strong residual confounding or construct overlap, which was not unexpected given the high covariance between a number of the different variables (Fig. 3).

### *Multivariate Associations With SRS*

The associations of a priori selected variables based on the literature with child SRS scores are shown in Table 2. Figure 3 demonstrates the high covariance among these variables with all other variables in the dataset. The six a priori selected variables did not show high intercorrelations, with the exception of maternal age with maternal education (*r* = 0.34), maternal age with parity (*r* = 0.29) and birth year with age at which the SRS was administered (*r* = −0.50). Not surprisingly, there was also a high correlation between birth weight and gestational age (*r* = 0.61), In our sample, the quadratic term for maternal age at birth showed a statistically significant association with the SRS score, so it was included in our analyses.

**Table 2:** Associations in the Generation R Study Between the Social Responsiveness Scale and Eight Variables That Are Typically Used as Covariates in Epidemiological Studies

Variable	Contrast^a^	*B*	CI 95% lower	CI 95% upper
Maternal age at birth (years)		−0.007	−0.008	−0.005
Maternal education	High vs. low	0.158	0.134	0.182
Maternal education	High vs. medium	0.053	0.035	0.071
Maternal ethnicity	NW vs. Dutch	−0.097	−0.116	−0.078
Maternal ethnicity	NW vs. OW	−0.102	−0.133	−0.071
Sex of the child	Boy vs. girl	−0.063	−0.079	−0.047
Parity		0.001	−0.015	0.018
Birth weight (g)		−1.2 × 10^−5^	−2.6 × 10^−5^	−5.1 × 10^−7^
Gestational age at birth (weeks)		−0.003	−0.008	0.001
Birth year	2002/3 vs. 2004	0.019	−0.002	0.039
Birth year	2002/3 vs. 2005/6	0.026	0.004	0.048

*B*: beta coefficient; CI: confidence interval; NW: nonwestern; OW: other western.

^a^ For categorical variables, the first element in the contrast is assigned a lower number compared to the second element in the contrast (i.e., a negative *B* with sex implies an inverse relationship and thus girls have lower SRS scores than boys).

**Figure 4:**
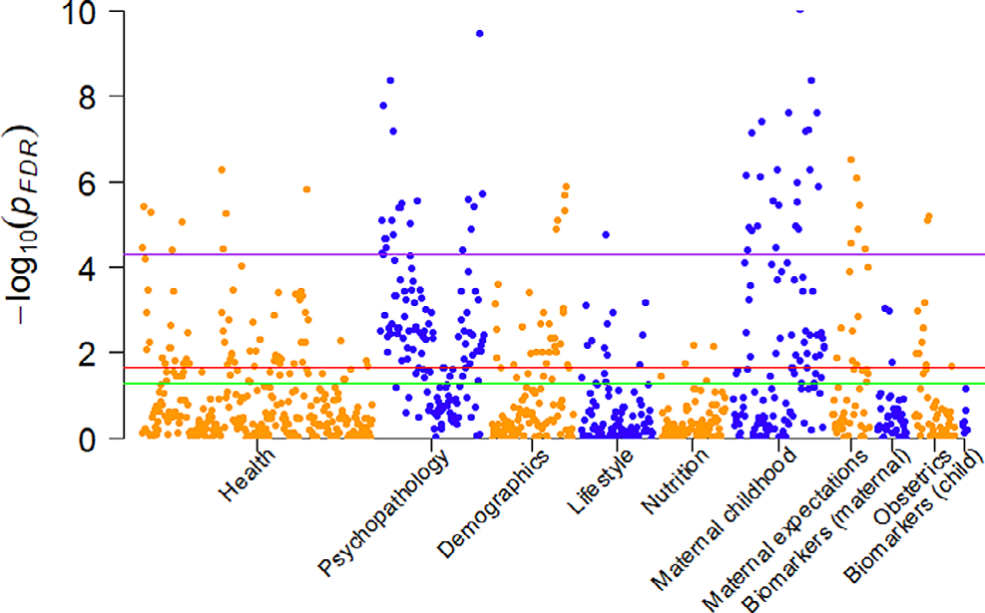
Manhattan plot of the false discovery rate (FDR)‐corrected minus log 10 *p*‐values of the adjusted iterative regression analysis. The analysis was adjusted for maternal age at birth, maternal education, maternal ethnicity, gender of the child, parity of the pregnancy, birth weight, gestational age at birth, and birth year. Each group of colored dots represents a domain. The green horizontal line marks the 0.05 uncorrected threshold. The red horizontal line marks the 0.05 FDR‐corrected threshold. The purple horizontal line marks the 0.05 Bonferroni‐corrected threshold.

The initial iterative regressions were corrected for these covariates and, as expected, nearly all variables had dramatically reduced effect estimates (Fig. 4). A total of 328 variables remained statistically significant after FDR correction in the discovery sample. The three domains with the highest proportion of statistically significant hits were parental psychopathology (95 out of 134 variables), parental health (83 out of 260) and family factors (64 out of 123). None of the variables included statistically significant quadratic terms.

### *Test Analysis*

The statistically significant hits in the discovery sample were subsequently analyzed within the test cohort. A total of 111 out of the 328 hits survived FDR correction and are shown in Table S2. Most statistically significant hits were derived from the domains parental psychopathology (44 out of 134), parental health (31 out of 260) and family factors (18 out of 54). Results of analyses using the six most common covariates that survive Bonferroni correction for multiple testing are shown in Table 3.

**Table 3:** Variables Shown That Predict the Later Development of Autistic Symptoms That Survive Bonferroni Correction in Both the Discovery and Test Sets Using the Six Most Common Covariates

Variable	Type of variable	Lowest to highest level	*n*	*B*	*B* int.	CIL	CIU	*p* FDR	*p* Bonf
Nervous (how often in the past month?)	Ordered categorical	Never to often	934	0.098	1.111	0.061	0.136	<0.0001	<0.0001
How good is your Dutch speaking?	Ordered categorical	Not at all to very good	891	−0.152	1.081	−0.214	−0.09	0.0001	0.0006
Obsessive–compulsive	Numerical		877	0.082	0.782	0.048	0.116	0.0001	0.0008
Feeling calm and contented in the past month?	Ordered categorical	Never to constantly	943	−0.133	0.832	−0.188	−0.078	0.0001	0.0008
Anxiety	Numerical		887	0.095	0.855	0.056	0.135	0.0001	0.001
Nervousness/shaking inside over the past week?	Ordered categorical	A little to continually	896	0.059	0.902	0.034	0.085	0.0001	0.002
Physical or emotional problems that hinder your activities over the past month?	Ordered categorical	Never to constantly	931	0.076	1.165	0.042	0.109	0.0004	0.004
My father praised me?	Ordered categorical	No never to yes always	827	−0.094	0.425	−0.137	−0.051	0.0005	0.0072
People in our family looked after each other	Numerical		885	−0.066	0.871	−0.096	−0.035	0.0005	0.008
Positive Symptom Total (PST)	Numerical	Not at all to continuous	823	0.004	0.802	0.002	0.006	0.0008	0.011
Feeling energetic in the past month	Ordered categorical	Never to constantly	928	−0.081	1.187	−0.119	−0.042	0.0008	0.01
Difficulty in making decisions in the past week?	Ordered categorical	A little to continually	900	0.048	0.95	0.025	0.071	0.001	0.016
Feeling happy in the past month?	Ordered categorical	Never to constantly	942	−0.127	0.885	−0.188	−0.065	0.001	0.02
Feeling down so that nothing could cheer you up	Ordered categorical	Never to constantly	941	0.081	0.782	0.041	0.121	0.001	0.029
Global Severity Index (GSI)	Numerical		888	0.106	0.83	0.053	0.16	0.002	0.0373
So down that nothing could cheer you up? How often past month?	Ordered categorical	Never to constantly	930	0.08	1.23	0.039	0.12	0.002	0.037

*Notes*: Variables that did not pass Bonferroni correction in the Dutch only sample, however, have been removed. All variables that survive FDR correction in both the discovery and test set for the full cohort after correction for the six most common covariates are presented in Table S2. All variables that survive FDR correction in both the discovery and test set for the Dutch‐only cohort after correction for the six most common covariates are presented in Table S3.

*B* int.: *B* intercept; CIL: lower confidence interval; CIU: upper confidence interval; *p* Bonf: Bonferroni corrected *p*‐value; *p* FDR: false discovery rate corrected *p*‐value.

While birth weight and gestational age at birth were commonly used as covariates in prior studies, but may also be on the causal pathway between exposure and autistic symptoms, we performed a sensitivity analysis also using these two variables as covariates. When rerunning these analyses including birth weight and gestational age at birth as covariates, there was considerable overlap in the findings. Only 16 variables were not significant in both the discovery and test set with and without the addition of these two variables as covariates. Twelve of these 16 variables that did not survive FDR correction when not controlling for birth weight and gestational age were variables primarily related to maternal depressive symptoms during pregnancy. The four variables that were statistically significant without birth weight and gestational age at birth as covariates fell into domains of anxiety and somatic complaints.

The discovery and test sets contained 37 and 16 children with a clinical diagnosis of ASD, respectively. To ensure that the associations were not driven by the clinical cases we reran the analyses excluding ASD cases. The results did not drastically change, with 307 statistically significant hits in the discovery cohort that survived correction for multiple testing.

### *Post Hoc Adjustment for Parental Psychopathology*

We found that symptoms of psychopathology in the parents were the most statistically significant predictors of the later development of autistic symptoms in children. Variables such as maternal anxiety, obsessive compulsive symptoms, positive symptoms, difficulties concentrating, emotional problems, and a measure of global psychopathology. All these variables passed the stringent Bonferroni correction in both the discovery and test cohorts (Table 4).

**Table 4:** The Number of Statistically Significant Variables for the E^n^WAS Analyses in Both the Discovery and Test Set for Each of the Different Analyses and Within Each of the Domains

	Discovery set			Test set		
	Total	*p* < 0.05	FDR	Total	*p* < 0.05	FDR
Total univariate analyses^a^	917			917	616	580
Total covaried analyses	911	404	328	328	171	111
Total additional psychopathology correction	910	279	156	156	57	23
Total Dutch only	902	299	179	179	61	29
Total Dutch only with additional psychopathology correction	899	137	28	28	4	0
*Domains (total covaried analyses)*						
Parental health	260	108	83	83	41	31
Parental psychology & psychopathology	134	106	95	95	59	44
Demographic characteristics	91	40	34	34	21	13
Parental prenatal lifestyle/life events	87	22	14	14	10	6
Parental exposures to nutrition/toxins	76	10	5	5	1	1
Family factors	123	75	64	64	29	18
Maternal expectations for the child	50	21	19	19	5	2
Maternal blood and urine biomarkers	40	8	3	3	0	0
Perinatal care and complications	43	13	11	11	5	1
Blood biomarkers for the child	7	1	0	0	0	0

^a^ The number of statistically significant variables were not calculated for the total univariate analyses in the test set due to the recognition of statistically significant confounding in the discovery set.

Due to the high number of statistically significant findings in the domain of parental psychopathology we added the maternal sum score of the BSI (a global measure of psychopathology) as a covariate to the regression models. In the discovery sample, a total of 156 variables showed statistically significant associations with the SRS score following FDR correction. Of these 156 variables, 23 variables were also significant following subsequent FDR correction in the test set (Table S4).

An overview of the total number of statistically significant variables for each of the analyses described above, including both the discovery and test cohorts, are shown in Table 4.

## Discussion

We utilized a large population‐based cohort of child development to study the relationship between multiple variables collected prospectively during prenatal and perinatal life with the later development of autistic traits. We found a wide array of variables obtained prospectively during prenatal and perinatal life that were associated with higher SRS scores measured on average 6 years after birth. Our initial univariate analyses resulted in statistically significant relationships in 580 out of 917 variables, even after FDR correction in both the discovery and test groups, which suggested high rates of residual confounding. When performing multiple linear regression analyses and including six of the most common covariates used in epidemiological studies of ASD, the number of statistically significant variables surviving FDR correction in the discovery cohort fell from 580 to 328 out of 912 variables. When testing these 328 variables in the test cohort, 111 variables remained statistically significant, of which the parental psychopathology domain showed the highest link to the future development of ASD. We then reran these analyses also correcting for birth weight and gestational age at birth, as these variables have often been used as covariates in prior studies (Table S1) and there was little change. Our primary analyses did not include these variables, as they could potentially be mediating variables [Lampi et al., 2012; Losh, Esserman, Anckarsäter, Sullivan, & Lichtenstein, 2012]. The majority of the variables were statistically significant using both six and eight covariates, with the exception being 16 variables in the domains of mood and anxiety symptoms. Future work should explore whether birth weight and gestational age at birth are mediators between maternal somatic complaints during pregnancy and autistic symptoms in offspring.

Interestingly, results from specific variables suggested that ethnic differences were driving some of the associations. Some of the statistically significant variables included how well the mothers can read, write, and communicate in Dutch. Not surprisingly, when we performed sensitivity analyses using data including only children with Dutch parents and grandparents, these variables were no longer statistically significant. It is possible that these ethnic differences might be related to how parents complete the SRS rather than actual ethnic differences related to autistic symptoms. For example, research has found that differences in maternal education, income, and ethnicity are associated with how mothers complete the SCQ [Rosenberg et al., 2018]. However, there is also evidence from US studies suggesting that differences in race and ethnicity may be related to the development of ASD, primarily in foreign‐born mothers of color [Becerra et al., 2014].

Hodges and colleagues [Hodge, Hoffman, & Sweeney, 2011] found that parents of children with ASD reported higher levels of obsessive–compulsive behaviors, interpersonal sensitivity, paranoid ideation and depression. They raised the question whether increased parental psychopathology was related to a genetic susceptibility or a result of the burden of caring for a child with ASD. Since we prospectively collected information on parental psychopathology during prenatal and perinatal life, our findings support the hypothesis of a genetic susceptibility to ASD as opposed to the burden of having a child with autistic traits.

An additional goal to develop a polyenvironmental risk score in the discovery group that could be then tested in the test set was not done in this study, primarily because of the high covariance structure of the data, coupled with combining biomarker and questionnaire data. Our approach of using a large number of individual multivariate analyses, with each individual variable analyzed separately with covariates is similar to performing multiple individual studies. What this approach does offer is to see not only which variables are related to the later development of autistic symptoms, but also which are not. Thus, while we present the information in a Manhattan‐like plot, we do not combine neither the variables to create summary scores.

After controlling for parental psychopathology and removing variables that might be related to ethnic differences, a series of variables remain that are related to family or interpersonal functioning (Tables 5 and S4). For example, two questions from the EMBU obtain information on how the mother's parents treated her during her childhood, such as praise and comfort from the father and family expectations and support. We found that mothers who reported less praise and comfort from her father when she was growing up, and a feeling of less expectations and love by her maternal parents was associated with greater autistic symptoms. Interestingly, this suggests that the risk for ASD might be observable in the social and emotional behaviors of previous generations. Given that ASD is highly heritable, this suggests that the genetic burden for ASD is also related to the spectrum of social behaviors in parents. However, we cannot make firm conclusions about the relationship between genetic factors and ASD symptoms in the family. Environmental and genetic variables collected across multiple generations, or adoption studies would be needed to confirm the generational transmission of symptoms within the autism spectrum.

**Table 5:** Variables Shown That Predict the Later Development of Autistic Symptoms That Survive FDR Correction in Both the Discovery and Test Sets Using the Six Most Common Covariates and the Global Severity Index (GSI)

Variable	Type of variable	Lowest to highest level	*n*	*B*	*B* int.	CIL	CIU	*p* FDR	*p* Bonf
Nervous, how often in the past month?	Ordered categorical	No/never to yes/always	934	0.084	0.56	0.044	0.123	0.001	0.006
My father praised me	Ordered categorical	Never to constantly	827	−0.086	−0.008	−0.13	−0.043	0.002	0.015
People in our family looked after each other	Ordered categorical	No/never to yes/always	885	−0.06	0.362	−0.09	−0.03	0.002	0.016
Someone in our family wanted me to achieve something	Ordered categorical	No/never to yes/always	888	−0.05	0.412	−0.081	−0.019	0.019	0.25
Someone in our family believed in me	Ordered categorical	No/never to yes/always	872	−0.047	0.322	−0.078	−0.016	0.026	0.44
I felt that I was loved	Ordered categorical	No/never to yes/always	887	−0.045	0.409	−0.075	−0.014	0.034	0.70
In general, how would you describe your health?	Ordered categorical	Poor to Excellent	928	−0.051	0.558	−0.089	−0.014	0.047	1
I felt that my father tried to comfort me	Ordered categorical	No/never to yes/always	808	−0.048	0.228	−0.083	−0.013	0.049	1

*Notes*: Variables that did not pass FDR correction in the Dutch only sample have been removed. All variables that survive FDR correction in the full cohort after correction for the six most common covariates and the GSI are presented in Table S4.

B int.: B intercept; CIL: lower confidence interval; CIU: upper confidence interval; *p* Bonf: Bonferroni corrected *p*‐value; *p* FDR: false discovery rate corrected *p*‐value.

While one goal was to use an approach to determine which variables may predict the later onset of autistic symptoms, our second was goal to perform a data‐driven approach to discover putative modifiable environmental variables associated with the later development of autistic symptoms. Our prior associations between environmental variables and autistic symptoms or ASD within the Generation R Study have included prenatal exposure to selective serotonin reuptake inhibitors [El Marroun et al., 2014], second trimester serum levels of vitamin D [Ars et al., 2016] and fatty acids [Steenweg‐de Graaff et al., 2016]. Thus, we asked the question whether any of the serum biomarkers included in our E^n^WAS had effect estimates that were greater than reported in our prior work. Four different fatty acids had effect estimates higher than our prior published biomarkers; these include ginkgolic acid (C15:1), linoleic acid (C18:1) linolelaidic acid (C18:2tt), and eicosenoic acid (C20:1). While these fatty acids reached statistically significance in the discovery sample, they were not statistically significant following multiple testing correction in the test sample; however, these could be interesting potential leads to explore further.

We primarily focused on presenting the effect estimates and p‐values for each variable and within each model. Whether a variable is statistically significant or not is heavily influenced by the distribution of the variable under study, the sparseness of cells in categorical variables, and the type of correction for multiple testing. We attempted to reduce spurious findings by utilizing a study with a large sample size, excluding individuals or variables in high rates of missing data or sparse variables, by the use of discovery and test sets, and by applying strict multiple testing correction.

While we could have included only summary measures for the questionnaire data into the E^n^WAS, we decided to include both summary measures and the individual items that made up the summary scores. While including only summary scores would have reduced the number of tests, this approach also involves assumptions that the summary measures are clustered in such a manner that they could better extract signals of emerging ASD in the offspring. We considered that it could be important to look at individual questions, perhaps even considering the possibility that a new questionnaire could be developed that captures individual items from different questionnaires, but with each question tapping some metric of later risk for ASD. Since we did multiple linear regression analyses, we ended up doing both and also corrected for all tests. In fact, there were four global measures that predicted those children who would have higher autistic symptoms (obsessive–compulsive, anxiety, positive symptoms, and the GSI) (Table 3).

While classically autism has been considered as a dichotomous disorder, the current description as ASD as a spectrum highlights a continuous nature of autistic symptoms [Constantino & Todd, 2003]. Similar claims have been made for depression [Angst, Merikangas, & Preisig, 1997] and psychosis [Landin‐Romero et al., 2016]. We attempted to study whether the continuous relationship holds in children with subclinical autistic features by excluding the clinically diagnosed ASD cases to see if the removal of those children would influence the results. We found that removal of the children with a diagnosis of ASD did not influence the results substantially, which suggests an extension of the relationships between autistic symptoms and environmental variables into subclinical symptoms. Interestingly, the remaining statistically significant variables were similar to those that have been associated with ASD in case–control designs [Gao et al., 2015]. As continuous outcomes can also lead to better powered statistical testing for small sample sizes [Bhandari, Lochner, & Tornetta 3rd., 2002], research on ASD may benefit from including instruments, that can measure autistic symptoms along a continuum, such as the SRS, SCQ, or the Autism Questionnaire [Adachi et al., 2018]. However, objective measures of autistic symptoms are important, since parents may have specific biases in how they report autistic symptoms based on their own demographic or clinical characteristics.

The relatively wide range of different statistically significant variables suggests that while each of these factors may play a small role in the development of autism, their combined effect may have larger consequences. Work directed toward the combination of different types of variables (i.e., questionnaire, serum or urine biomarkers) in the generation of “polyenvironmental risk score” would be beneficial. We did not find one specific environmental variable that accounted for a large amount of the autistic symptoms, thus it may be autistic symptoms are related to an interplay between the different environmental variables. More research is needed to identify the important interplay between the variables that consistently contribute to the risk of ASD, as well as determining whether preventative measures can modify these variables to reduce the risk. In addition, with the advent of statistical learning techniques and the emphasis on prediction models, a data‐wide approach could enable the construction of an optimal ASD risk score collected prospectively during prenatal life that can be used to identify mothers who are at highest potential risk. These families could potentially benefit from preventative measures.

There are several strengths of this study, including the large sample size, a prospective population‐based sample, the use of both discovery and test samples, testing the relationship of multiple variables with autistic symptoms, and the comparison between different potential variables and biomarkers. However, there are also several limitations to the study. The number of children who have a diagnosis of ASD is not large. Thus, we lacked adequate power to utilize a discovery and test sample to evaluate children with an ASD diagnosis. A second major limitation is that we lacked multiple informant measures for autistic symptoms. The use of multiple informants could have been used to assess the role of shared method variance bias [Ringoot et al., 2015]. Thus, it is possible that the individual characteristics in how the mother rated questionnaires about herself would show the same patterns in who she rated her child's autistic symptoms. In addition, exposures are changing over time, even in relatively short period of pregnancy, which can result in reverse causality bias.

It has been suggested that an E^n^WAS approach may lead to high rates of false positives [Siroux, Agier, & Slama, 2016]. However, by using discovery and test sets, these errors can be minimized. By performing FDR correction for both the discovery and replication data sets, we have effectively controlled for any chance related findings due to the number of statistical tests performed. This is because in the case of random variables that are normally distributed, the FDR approach (which would also be equivalent to using a family‐wise error approach) would result in an expected 5% chance of having just one false positive from the 920 tests [T. White, van der Ende, & Nichols, 2019]. For tests that are significant in the discovery set, using the test sample, in which FDR is also applied, the discovery and test sample combined would have a 0.25% chance of having one false positive in 920 tests. Thus, our EnWAS approach is not highly susceptible to false positives due to chance findings, but may be due to other aspects related to the nature of the data itself. The three most likely possibilities are i.) the findings are true and parental psychopathology and health factors are related to the later development of child autistic symptoms; ii.) there is residual confounding due to variables that we were unable to measure; and iii.) there is bias as a result of shared rater bias variance. Furthermore, a limitation is the lack of intervariable estimation; because only one determinant per model was considered.

While we use the term “environmental,” we recognize that some variables, while not genetic data, are likely driven by genetic factors. Higher rates of maternal psychopathology contributes to psychopathology in the offspring via genetic susceptibility [Agha, Zammit, Thapar, & Langley, 2017]. Further, biomarkers such as vitamin D likely also have genetic underpinnings related to uptake and metabolism [Matyjaszek‐Matuszek, Lenart‐Lipinska, & Wozniakowska, 2015], thus there is often a fuzzy border between environmental and genetic factors. Another limitation of the dataset is the low number of complete cases if we consider all 920 variables. Missing data is typically not at random and thus can bias the results. Additional limitations were that some of the categorical variables had selections with low frequency and these variables were excluded due to inadequate power to detect differences. Further, the combination of environmental variables have different challenges than the approaches used for genome‐wide association studies, due to the often high covariance between environmental variables coupled with some variables having different levels of weights. We did not parameterize the biomarkers, but rather, the biomarkers were analyzed as continuous variables. This approach may be less likely to identify nonlinear relationships between variables and could be another limitation of this study. However, we did explore quadratic relationships between the biomarkers and autistic symptoms. Finally, there is high covariance between variables used in our study and while we corrected for six variables commonly used in epidemiological studies of ASD, the possibility of hidden confounding is certainly possible.

In conclusion, we performed an environmental‐wide association study of autistic traits using variables collected prospectively during prenatal and perinatal life and found a number of variables that predicted higher autistic symptoms during childhood. No one variable towered above the others, suggesting that it may be the interplay between these variables that is associated with emerging autistic symptoms, Alternatively, it may be driven more by genetic [Taylor et al., 2020] or stochastic [T. J. H. White, 2019] events than environmental factors. Further research should explore whether the combination of multiple environmental variables, each having a small effect contributes to the emergence of autistic symptoms. If so, the creation of a “polyenvironmental risk score” would provide greater prediction of emerging autistic symptoms.

## Conflict of Interest

The author declares that there is no conflict of interest that could be perceived as prejudicing the impartiality of the research reported.

## Supporting information


**Figure S1**. (A) Distribution of the SRS scores in Generation R Study (*histogram*); and (B) SRS scores between those with and without a clinical diagnosis of ASD (*boxplot*)
**Table S1**. Main characteristics of Replication group, no Global Symptom Inventory (GSI) and all children
**Table S2**. Main results in replication group, not corrected for the Global Symptom Inventory (GSI)
**Table S3**. Main results in the Dutch sample (discovery + replication combined), not corrected for GSI
**Table S4**. Main results in replication group, corrected for GSI
