# Supplementary material for: Environment‐Wide Association Study (EnWAS) of Prenatal and Perinatal Factors Associated With Autistic Traits: A Population‐Based Study
Source: Autism Res. 2020 Aug 23;13(9):1582–600. doi: 10.1002/aur.2372 (PMC7540497; doi:10.1002/aur.2372)
Supplement: Supplementary file 1 — Figure S1. (A) Distribution of the SRS scores in Generation R Study (histogram); and (B) SRS scores between those with and without a clinical diagnosis of ASD (boxplot) Table S1. Main characteristics of Replication group, no Global Symptom Inventory (GSI) and all children Table S2. Main results in replication group, not corrected for the Global Symptom Inventory (GSI) Table S3. Main results in the Dutch sample (discovery + replication combined), not corrected for GSI Table S4. Main results in replication group, corrected for GSI [file AUR-13-1582-s001.docx]

**Supplementary figure 1**- (A) Distribution of the SRS scores in Generation R Study (*histogram*); and (B) SRS scores between those with and without a clinical diagnosis of ASD (*boxplot*).

*
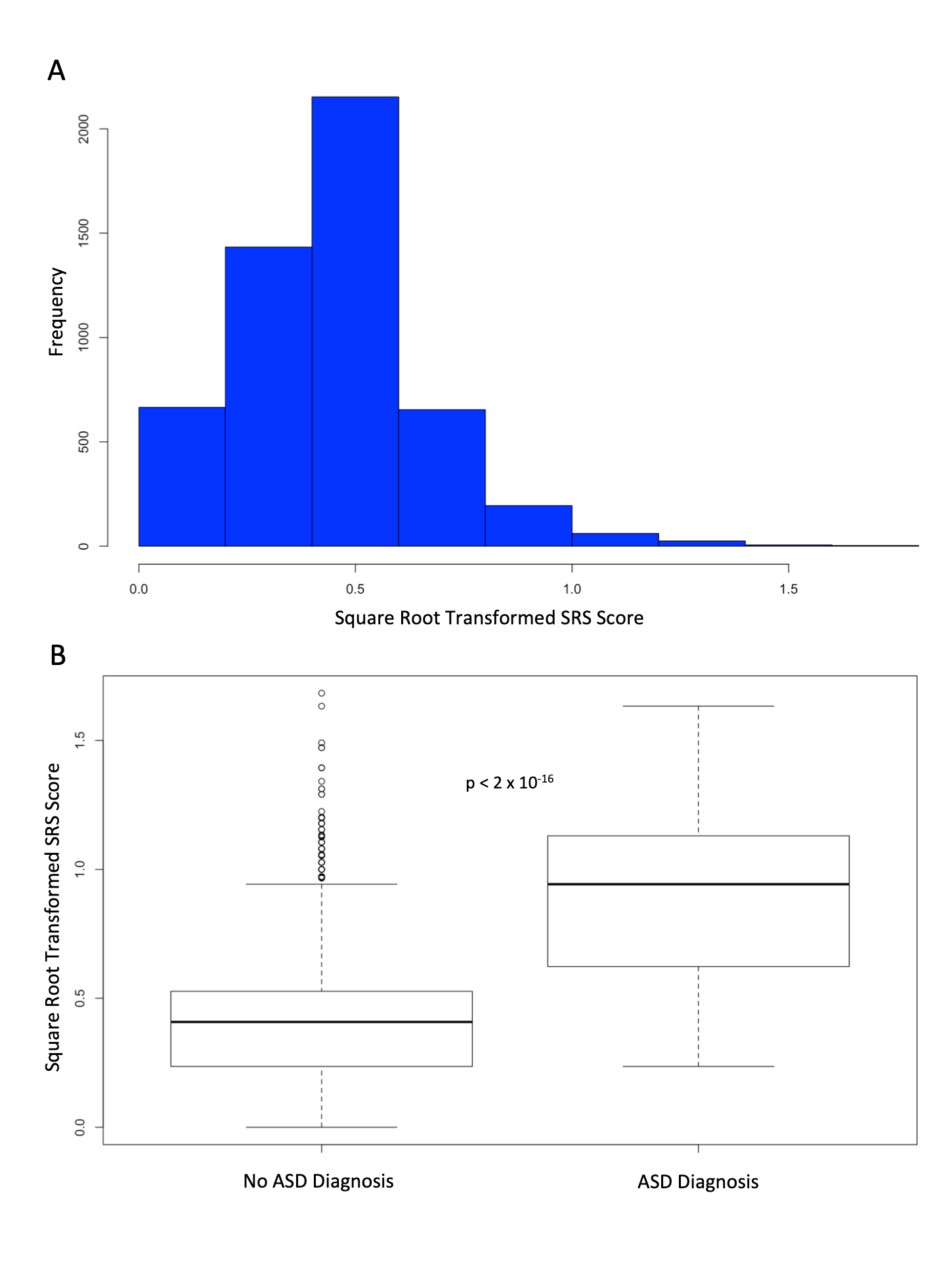
*

**Supplementary Table 1- Main characteristics of Replication group, no Global Symptom Inventory (GSI) and all children**

| Domain | Variable | Sample size | Beta | CIL | CIU | P-value | P-FDR | P-  Bonferroni | After GSI correction |
| --- | --- | --- | --- | --- | --- | --- | --- | --- | --- |
| 1 | Generally, how would you describe your health? | 944 | -0.055 | -0.093 | -0.017 | 0.004 | 0.024 | 1.00 | FALSE |
| 1 | Restricted by your health or the pregnancy moderate exercise? | 933 | 0.056 | 0.017 | 0.096 | 0.005 | 0.026 | 1.00 | FALSE |
| 1 | Because of your health unable to carry out activities past month | 925 | 0.045 | 0.014 | 0.076 | 0.005 | 0.026 | 1.00 | FALSE |
| 1 | How often has physical health or emotional problems hindered? | 941 | 0.058 | 0.024 | 0.092 | 0.001 | 0.008 | 0.27 | FALSE |
| 1 | Reaching for something on a high shelf affecting your daily life | 669 | 0.065 | 0.022 | 0.109 | 0.003 | 0.020 | 1.00 | FALSE |
| 1 | have you suffered from pain in muscles or joints past 2 months? | 924 | 0.052 | 0.022 | 0.082 | 0.001 | 0.008 | 0.27 | FALSE |
| 1 | Have you suffered from pain in lower abdomen past 2 months? | 932 | 0.048 | 0.018 | 0.078 | 0.002 | 0.012 | 0.57 | FALSE |
| 1 | Urination pain past 2 months? | 917 | 0.082 | 0.02 | 0.144 | 0.009 | 0.037 | 1.00 | FALSE |
| 1 | In general, how would you describe your health? | 928 | -0.065 | -0.101 | -0.029 | 0.000 | 0.006 | 0.15 | FALSE |
| 1 | Moderate exercise restricted by health or pregnancy now | 928 | 0.055 | 0.017 | 0.092 | 0.004 | 0.023 | 1.00 | FALSE |
| 1 | Going upstairs restricted by health or pregnancy now | 923 | 0.055 | 0.017 | 0.093 | 0.004 | 0.024 | 1.00 | FALSE |
| 1 | Unable to do work or activity by physical health past month | 925 | 0.053 | 0.024 | 0.083 | 0.000 | 0.006 | 0.15 | FALSE |
| 1 | Physical or emotional problem hinder your activities past month | 931 | 0.076 | 0.042 | 0.109 | 0.000 | 0.000 | 0.00 | TRUE |
| 1 | Tiredness past 3 months | 924 | 0.059 | 0.018 | 0.1 | 0.005 | 0.026 | 1.00 | FALSE |
| 1 | Pain in upper abdomen past 3 months | 914 | 0.04 | 0.008 | 0.072 | 0.014 | 0.047 | 1.00 | FALSE |
| 1 | Pain in lower abdomen past 3 months | 920 | 0.048 | 0.018 | 0.078 | 0.002 | 0.012 | 0.57 | FALSE |
| 1 | Burning feeling with urination/ urethra past 3 months | 918 | 0.071 | 0.015 | 0.128 | 0.014 | 0.047 | 1.00 | FALSE |
| 1 | How would you describe your health in general? | 879 | -0.056 | -0.096 | -0.016 | 0.006 | 0.027 | 1.00 | FALSE |
| 1 | Nervousness or shaking inside past week? | 896 | 0.059 | 0.034 | 0.085 | 0.000 | 0.000 | 0.00 | FALSE |
| 1 | Dizziness past week? | 898 | 0.03 | 0.006 | 0.053 | 0.013 | 0.046 | 1.00 | FALSE |
| 1 | Having little appetite past week? | 899 | 0.042 | 0.014 | 0.07 | 0.003 | 0.019 | 1.00 | FALSE |
| 1 | Nausea or an upset stomach past week? | 894 | 0.031 | 0.007 | 0.054 | 0.010 | 0.038 | 1.00 | FALSE |
| 1 | Feeling physically weak past week? | 899 | 0.027 | 0.005 | 0.049 | 0.015 | 0.048 | 1.00 | FALSE |
| 1 | Feeling tense past week? | 897 | 0.038 | 0.016 | 0.061 | 0.001 | 0.009 | 0.33 | FALSE |
| 1 | Has he ever been tested for HIV_AIDS_biological father? | 892 | -0.091 | -0.157 | -0.025 | 0.007 | 0.032 | 1.00 | FALSE |
| 1 | Height mother | 983 | -0.003 | -0.005 | -0.001 | 0.009 | 0.036 | 1.00 | FALSE |
| 1 | Pregnancy hypertension mother or sisters | 878 | -0.082 | -0.138 | -0.026 | 0.004 | 0.024 | 1.00 | FALSE |
| 2 | As a result of emotional problems unable to carry out activities | 925 | 0.073 | 0.018 | 0.129 | 0.010 | 0.038 | 1.00 | FALSE |
| 2 | Have you felt very nervous past month? | 940 | 0.057 | 0.019 | 0.094 | 0.003 | 0.019 | 1.00 | FALSE |
| 2 | Have you felt so down that nothing could cheer you up past month? | 941 | 0.081 | 0.041 | 0.121 | 0.000 | 0.001 | 0.03 | FALSE |
| 2 | Have you felt calm and contented past month? | 943 | -0.133 | -0.188 | -0.078 | 0.000 | 0.000 | 0.00 | TRUE |
| 2 | Have you felt very energetic past month? | 941 | -0.051 | -0.091 | -0.011 | 0.012 | 0.044 | 1.00 | FALSE |
| 2 | Have you felt down and depressed past month? | 936 | 0.073 | 0.035 | 0.111 | 0.000 | 0.002 | 0.05 | FALSE |
| 2 | Have you felt happy past month? | 942 | -0.127 | -0.188 | -0.065 | 0.000 | 0.001 | 0.02 | TRUE |
| 2 | Anxiety | 887 | 0.095 | 0.056 | 0.135 | 0.000 | 0.000 | 0.00 | FALSE |
| 2 | Achieved less emotional problems past month | 928 | 0.068 | 0.02 | 0.116 | 0.006 | 0.027 | 1.00 | FALSE |
| 2 | No work or activities emotional problems past month | 924 | 0.079 | 0.028 | 0.131 | 0.003 | 0.018 | 0.94 | FALSE |
| 2 | Prevented from normal activity due to pain past month | 929 | 0.054 | 0.015 | 0.093 | 0.006 | 0.029 | 1.00 | FALSE |
| 2 | Nervous how often past month | 934 | 0.098 | 0.061 | 0.136 | 0.000 | 0.000 | 0.00 | TRUE |
| 2 | So down that nothing could cheer you up how often past month | 930 | 0.08 | 0.039 | 0.12 | 0.000 | 0.002 | 0.04 | FALSE |
| 2 | Calm or contented how often past month | 931 | -0.11 | -0.164 | -0.056 | 0.000 | 0.001 | 0.03 | FALSE |
| 2 | Energetic how often past month | 928 | -0.081 | -0.119 | -0.042 | 0.000 | 0.001 | 0.01 | TRUE |
| 2 | How often did you feel very nervous past month? | 878 | 0.066 | 0.027 | 0.104 | 0.001 | 0.008 | 0.27 | FALSE |
| 2 | How often did you feel down and depressed past month? | 870 | 0.049 | 0.01 | 0.089 | 0.014 | 0.047 | 1.00 | FALSE |
| 2 | How often hindering of social activities past month? | 876 | 0.043 | 0.008 | 0.078 | 0.016 | 0.050 | 1.00 | FALSE |
| 2 | Feeling that others are the cause of your problems past week? | 898 | 0.051 | 0.017 | 0.086 | 0.003 | 0.019 | 1.00 | FALSE |
| 2 | Feeling anxious in open spaces or on the street past week? | 896 | 0.062 | 0.014 | 0.109 | 0.010 | 0.040 | 1.00 | FALSE |
| 2 | Feeling that most people cannot be trusted past week? | 896 | 0.042 | 0.009 | 0.076 | 0.014 | 0.047 | 1.00 | FALSE |
| 2 | Suddenly getting a fright or feeling anxious past week? | 896 | 0.047 | 0.014 | 0.08 | 0.006 | 0.027 | 1.00 | FALSE |
| 2 | Feeling alone; even when with other people past week? | 896 | 0.033 | 0.006 | 0.061 | 0.016 | 0.050 | 1.00 | FALSE |
| 2 | Feeling impeded when doing all kinds of things past week? | 892 | 0.032 | 0.007 | 0.057 | 0.012 | 0.045 | 1.00 | FALSE |
| 2 | Feeling that others are watching you past week? | 896 | 0.042 | 0.009 | 0.075 | 0.013 | 0.045 | 1.00 | FALSE |
| 2 | Difficulty in making decisions past week? | 900 | 0.048 | 0.025 | 0.071 | 0.000 | 0.001 | 0.02 | FALSE |
| 2 | Difficulty in concentrating past week? | 897 | 0.03 | 0.008 | 0.052 | 0.009 | 0.036 | 1.00 | FALSE |
| 2 | Thinking about death or dying past week? | 895 | 0.042 | 0.009 | 0.075 | 0.013 | 0.046 | 1.00 | FALSE |
| 2 | Feeling uncomfortable in crowds past week? | 897 | 0.064 | 0.025 | 0.102 | 0.001 | 0.010 | 0.39 | FALSE |
| 2 | Feeling that others underestimate your true worth past week? | 895 | 0.043 | 0.014 | 0.071 | 0.003 | 0.019 | 1.00 | FALSE |
| 2 | Feeling so restless that you cannot sit still past week? | 897 | 0.047 | 0.018 | 0.077 | 0.001 | 0.012 | 0.50 | FALSE |
| 2 | Have you ever had a period in which you were anxious or worried? | 888 | -0.128 | -0.228 | -0.028 | 0.012 | 0.044 | 1.00 | FALSE |
| 2 | Have you ever had a period in which you felt down or depressed? | 889 | -0.151 | -0.266 | -0.035 | 0.011 | 0.041 | 1.00 | FALSE |
| 2 | I sometimes feel really useless | 877 | 0.038 | 0.013 | 0.062 | 0.003 | 0.018 | 0.91 | FALSE |
| 2 | I wish I could feel more respect for myself | 871 | 0.042 | 0.017 | 0.068 | 0.001 | 0.009 | 0.34 | FALSE |
| 2 | Global Severity Index (GSI) | 888 | 0.106 | 0.053 | 0.16 | 0.000 | 0.002 | 0.04 | FALSE |
| 2 | Hostility | 883 | 0.066 | 0.021 | 0.11 | 0.004 | 0.022 | 1.00 | FALSE |
| 2 | We avoid talking about our worries and problems family now | 869 | 0.075 | 0.038 | 0.112 | 0.000 | 0.001 | 0.02 | FALSE |
| 2 | Obsessive-Compulsive | 877 | 0.082 | 0.048 | 0.116 | 0.000 | 0.000 | 0.00 | FALSE |
| 2 | Paranoid Ideation | 886 | 0.071 | 0.031 | 0.111 | 0.001 | 0.007 | 0.20 | FALSE |
| 2 | Positive Symptom Total (PST) | 823 | 0.004 | 0.002 | 0.006 | 0.000 | 0.001 | 0.01 | FALSE |
| 3 | Understanding Dutch | 766 | -0.108 | -0.193 | -0.022 | 0.014 | 0.05 | 1.00 | FALSE |
| 3 | Writing Dutch | 762 | -0.082 | -0.147 | -0.016 | 0.015 | 0.05 | 1.00 | FALSE |
| 3 | Feel part of the Dutch culture agreement | 881 | -0.085 | -0.133 | -0.036 | 0.001 | 0.01 | 0.24 | TRUE |
| 3 | Do you spend free time outside the home? | 861 | -0.053 | -0.096 | -0.01 | 0.015 | 0.05 | 1.00 | FALSE |
| 3 | Do you spend leisure time with Dutch people? | 853 | -0.086 | -0.133 | -0.039 | 0.000 | 0.00 | 0.12 | TRUE |
| 3 | Taking part in traditional Dutch celebrations/festivals | 856 | -0.058 | -0.106 | -0.011 | 0.016 | 0.05 | 1.00 | FALSE |
| 3 | Threatened or attacked due to ethnicity agreement | 709 | 0.074 | 0.017 | 0.13 | 0.010 | 0.04 | 1.00 | FALSE |
| 3 | Education father four scales | 814 | -0.058 | -0.094 | -0.022 | 0.002 | 0.01 | 0.52 | TRUE |
| 3 | Immigration status | 978 | 0.073 | 0.028 | 0.119 | 0.002 | 0.01 | 0.56 | TRUE |
| 3 | Net income household | 919 | -0.113 | -0.154 | -0.072 | 0.000 | 0.00 | 0.00 | TRUE |
| 3 | How good is your Dutch speaking? | 891 | -0.152 | -0.214 | -0.09 | 0.000 | 0.00 | 0.00 | TRUE |
| 3 | How good is your Dutch reading? | 888 | -0.144 | -0.208 | -0.08 | 0.000 | 0.00 | 0.00 | TRUE |
| 3 | How good is your Dutch writing? | 888 | -0.144 | -0.2 | -0.087 | 0.000 | 0.00 | 0.00 | TRUE |
| 4 | Difficulties in contact with others past year? | 861 | 0.12 | 0.049 | 0.192 | 0.001 | 0.01 | 0.33 | FALSE |
| 4 | Regular purchase of new clothes_possesion | 925 | 0.054 | 0.016 | 0.092 | 0.005 | 0.03 | 1.00 | FALSE |
| 4 | Car (or lease car) _possession | 925 | 0.059 | 0.012 | 0.106 | 0.015 | 0.05 | 1.00 | FALSE |
| 4 | Having friends or family round to eat 1 or more times a month | 929 | 0.057 | 0.016 | 0.099 | 0.007 | 0.03 | 1.00 | FALSE |
| 4 | Holiday away from home for one week or more each year | 923 | 0.085 | 0.035 | 0.134 | 0.001 | 0.01 | 0.29 | TRUE |
| 4 | Sllbom | 870 | 0.008 | 0.002 | 0.014 | 0.006 | 0.03 | 1.00 | FALSE |
| 5 | Known folic acid used and 100% certainly perceptional | 791 | -0.06 | -0.109 | -0.012 | 0.014 | 0.05 | 1.00 | FALSE |
| 6 | Having a car or lease car? | 839 | -0.056 | -0.1 | -0.012 | 0.013 | 0.045 | 1.000 | FALSE |
| 6 | Did you go on holiday with the family one week or more a year? | 842 | -0.066 | -0.109 | -0.022 | 0.003 | 0.019 | 1.000 | FALSE |
| 6 | Leisure equipment such as sports equipment or bicycle? | 842 | -0.087 | -0.147 | -0.027 | 0.004 | 0.024 | 1.000 | FALSE |
| 6 | My father praised me | 827 | -0.094 | -0.137 | -0.051 | 0.000 | 0.001 | 0.007 | TRUE |
| 6 | My mother praised me | 851 | -0.064 | -0.109 | -0.02 | 0.005 | 0.026 | 1.000 | FALSE |
| 6 | I think my father tried to make my childhood interesting | 827 | -0.058 | -0.094 | -0.022 | 0.002 | 0.013 | 0.613 | FALSE |
| 6 | I think my mother tried to make my childhood interesting | 861 | -0.06 | -0.098 | -0.022 | 0.002 | 0.015 | 0.730 | FALSE |
| 6 | I had the feeling that my father tried to comfort me | 808 | -0.06 | -0.094 | -0.025 | 0.001 | 0.008 | 0.246 | TRUE |
| 6 | My father treated me in such a way that I felt ashamed | 816 | 0.088 | 0.022 | 0.154 | 0.009 | 0.036 | 1.000 | FALSE |
| 6 | People in my family encouraged me to achieve things | 883 | -0.042 | -0.074 | -0.01 | 0.009 | 0.038 | 1.000 | FALSE |
| 6 | There was someone in our family who made me feel special | 884 | -0.049 | -0.083 | -0.014 | 0.005 | 0.026 | 1.000 | FALSE |
| 6 | Someone in our family wanted me to achieve something | 888 | -0.054 | -0.085 | -0.023 | 0.001 | 0.007 | 0.222 | TRUE |
| 6 | I felt that I was loved | 887 | -0.054 | -0.085 | -0.024 | 0.001 | 0.006 | 0.174 | TRUE |
| 6 | People in our family felt a bond between each other | 869 | -0.046 | -0.077 | -0.014 | 0.005 | 0.026 | 1.000 | FALSE |
| 6 | People in our family looked after each other | 885 | -0.066 | -0.096 | -0.035 | 0.000 | 0.001 | 0.008 | TRUE |
| 6 | Someone in our family believed in me | 872 | -0.055 | -0.086 | -0.024 | 0.000 | 0.006 | 0.158 | TRUE |
| 6 | Our family was a source of strength and support | 883 | -0.041 | -0.073 | -0.009 | 0.012 | 0.044 | 1.000 | FALSE |
| 7 | Mothers prefer their babies not to fidget and wriggle so much | 867 | 0.084 | 0.033 | 0.136 | 0.001 | 0.012 | 0.477 | TRUE |
| 7 | Mothers should not insist that children do what they were asked | 728 | 0.065 | 0.016 | 0.114 | 0.009 | 0.036 | 1.000 | FALSE |
| 9 | General satisfaction about the obstetric care so far | 936 | -0.082 | -0.143 | -0.02 | 0.009 | 0.037 | 1.000 | FALSE |
| 9 | Rank Child Mother | 986 | -0.074 | -0.129 | -0.02 | 0.008 | 0.034 | 1.000 | FALSE |

Domain 1 = Parental health; Domain 2 = Parental psychology and psychopathology; Domain 3 = Demographics of parents and grandparents; Domain 4 = Parental lifestyle and life events; Domain 5 = Parental exposure to nutrition, toxins and other chemicals; Domain 6 = Family; Domain 7 = Maternal expectations regarding the child; Domain 9 = Perinatal complications and obstetrics.

P-FDR = P- False discovery rate.

GSI correction = Global severity index

**Supplementary Table 2: Main results in replication group, not corrected for the Global Symptom Inventory (GSI)**

| **Variable** | **Type** |  | **n** | **B** | **CIL** | **CIU** | **p FDR** | **p Bonf** |
| --- | --- | --- | --- | --- | --- | --- | --- | --- |
| Generally how would you describe your health? | ordered | 'Poor' to 'Very good' | 944 | -0.055 | -0.093 | -0.017 | 0.0239 | 1.0000 |
| Restricted by your health or the pregnancy_moderate exercise? | ordered | 'Not restricted' to 'Severely restricted' | 933 | 0.056 | 0.017 | 0.096 | 0.0260 | 1.0000 |
| Because of your health_unable to carry out activities_past month | unordered | 'No' to 'Yes' | 925 | 0.045 | 0.014 | 0.076 | 0.0258 | 1.0000 |
| As a result of emotional problems_unable to carry out activities | unordered | 'No' to 'Yes' | 925 | 0.073 | 0.018 | 0.129 | 0.0381 | 1.0000 |
| As a result of emotional problems_unable to carry out activities | ordered | 'Never' to 'Often' | 940 | 0.057 | 0.019 | 0.094 | 0.0194 | 1.0000 |
| General satisfiction about the obstetric care so far | ordered | 'Very dissatifsfied' to 'Very satisfied' | 936 | -0.082 | -0.143 | -0.02 | 0.0374 | 1.0000 |
| Have you felt so down that nothing could cheer you up_past month | ordered | 'Never' to 'Often' | 941 | 0.081 | 0.041 | 0.121 | 0.0015 | 0.0295 |
| Have you felt calm and contented_past month? | ordered | 'Never' to 'Often' | 943 | -0.133 | -0.188 | -0.078 | 0.0001 | 0.0009 |
| Have you felt very energetic_past month? | ordered | 'Never' to 'Often' | 941 | -0.051 | -0.091 | -0.011 | 0.0435 | 1.0000 |
| Have you felt down and depressed_past month? | ordered | 'Never' to 'Often' | 936 | 0.073 | 0.035 | 0.111 | 0.0022 | 0.0514 |
| Have you felt happy_past month? | ordered | 'Never' to 'Often' | 942 | -0.127 | -0.188 | -0.065 | 0.0012 | 0.0210 |
| How often has physical health or emotional problems hindered? | ordered | 'Never' to 'Often' | 941 | 0.058 | 0.024 | 0.092 | 0.0077 | 0.2683 |
| Reaching for something on a high shelf_affecting your daily life | ordered | 'No difficulty' to 'A lot of difficulty' | 669 | 0.065 | 0.022 | 0.109 | 0.0205 | 1.0000 |
| Have you suffered from pain in muscles or joints_past 2 months? | ordered | 'Never' to 'Few days a week' | 924 | 0.052 | 0.022 | 0.082 | 0.0077 | 0.2662 |
| Have you suffered from pain in lower abdomen_past 2 months? | ordered | 'Never' to 'Few days a week' | 932 | 0.048 | 0.018 | 0.078 | 0.0121 | 0.5653 |
| urination pain_past 2 months? | unordered | 'No' to 'Yes' | 917 | 0.082 | 0.02 | 0.144 | 0.0374 | 1.0000 |
| BSI: Anxiety | numerical |  | 887 | 0.095 | 0.056 | 0.135 | 0.0001 | 0.0010 |
| In general_how would you describe your health | ordered | 'Poor' to 'Very good' | 928 | -0.065 | -0.101 | -0.029 | 0.0058 | 0.1480 |
| Moderate exercise_restricted by health or pregnancy now | ordered | 'Not restricted' to 'Severely restricted' | 928 | 0.055 | 0.017 | 0.092 | 0.0230 | 1.0000 |
| Going upstairs_restricted by health or pregnancy now | ordered | 'Not restricted' to 'Severely restricted' | 923 | 0.055 | 0.017 | 0.093 | 0.0239 | 1.0000 |
| Unable to do work or activity_by physical health_past month | unordered | 'No' to 'Yes' | 925 | 0.053 | 0.024 | 0.083 | 0.0058 | 0.1502 |
| Achieved less_emotional problems_past month | unordered | 'No' to 'Yes' | 928 | 0.068 | 0.02 | 0.116 | 0.0272 | 1.0000 |
| No work or activities_emotional problems_past month | unordered | 'No' to 'Yes' | 924 | 0.079 | 0.028 | 0.131 | 0.0181 | 0.9406 |
| Prevented from normal activity due to pain_past month | ordered | 'Not at all' to 'A lot' | 929 | 0.054 | 0.015 | 0.093 | 0.0292 | 1.0000 |
| Nervous_how often_past month | ordered | 'Never' to 'Often' | 934 | 0.098 | 0.061 | 0.136 | 3.0525e-05 | 9.1575e-05 |
| So down_that nothing could cheer you up_how often past month | ordered | 'Never' to 'Often' | 930 | 0.08 | 0.039 | 0.12 | 0.0017 | 0.0377 |
| Calm or contented_how often_past month | ordered | 'Never' to 'Often' | 931 | -0.11 | -0.164 | -0.056 | 0.0014 | 0.0264 |
| Energetic_how often_past month | ordered | 'Never' to 'Often' | 928 | -0.081 | -0.119 | -0.042 | 0.0008 | 0.0123 |
| Physical or emotional problem hinder your activities_past month | ordered | 'Never' to 'Often' | 931 | 0.076 | 0.042 | 0.109 | 0.0004 | 0.0044 |
| Tiredness_past 3 months | ordered | 'Never' to 'Few days a week' | 924 | 0.059 | 0.018 | 0.1 | 0.0256 | 1.0000 |
| Pain in upper abdomen_past 3 months | ordered | 'Never' to 'Few days a week' | 914 | 0.04 | 0.008 | 0.072 | 0.0469 | 1.0000 |
| Pain in lower abdomen_past 3 months | ordered | 'Never' to 'Few days a week' | 920 | 0.048 | 0.018 | 0.078 | 0.0121 | 0.5685 |
| Burning feeling with urination/ urethra_past 3 months | unordered | 'No' to 'Yes' | 918 | 0.071 | 0.015 | 0.128 | 0.0469 | 1.0000 |
| ow would you describe your health in general | ordered | 'Poor' to 'Very good' | 879 | -0.056 | -0.096 | -0.016 | 0.0272 | 1.0000 |
| Difficulties in contact with others_past year? | ordered | 'No' to 'Serious' | 861 | 0.12 | 0.049 | 0.192 | 0.0086 | 0.3266 |
| How often did you feel very nervous_past month | ordered | 'Never' to 'Often' | 878 | 0.066 | 0.027 | 0.104 | 0.0077 | 0.2685 |
| How often did you feel down and depressed_past month | ordered | 'Never' to 'Often' | 870 | 0.049 | 0.01 | 0.089 | 0.0469 | 1.0000 |
| How often hindering of social activities_past month | ordered | 'Never' to 'Often' | 876 | 0.043 | 0.008 | 0.078 | 0.0495 | 1.0000 |
| BSI: nervousness or shaking inside_past week? | ordered | 'Not at all' to 'A little' | 896 | 0.059 | 0.034 | 0.085 | 0.0002 | 0.0017 |
| BSI: dizziness_past week? | ordered | 'Not at all' to 'A little' | 898 | 0.03 | 0.006 | 0.053 | 0.0465 | 1.0000 |
| BSI: feeling that others are the cause of your problems_past week? | ordered | 'Not at all' to 'A little' | 898 | 0.051 | 0.017 | 0.086 | 0.0194 | 1.0000 |
| BSI: feeling anxious in open spaces or on the street_past week? | ordered | 'Not at all' to 'A little' | 896 | 0.062 | 0.014 | 0.109 | 0.0400 | 1.0000 |
| BSI: feeling that most people cannot be trusted_past week? | ordered | 'Not at all' to 'A little' | 896 | 0.042 | 0.009 | 0.076 | 0.0469 | 1.0000 |
| BSI: having little appetite_past week? | ordered | 'Not at all' to 'A little' | 899 | 0.042 | 0.014 | 0.07 | 0.0194 | 1.0000 |
| BSI: suddenly getting a fright or feeling anxious_past week? | ordered | 'Not at all' to 'A little' | 896 | 0.047 | 0.014 | 0.08 | 0.0268 | 1.0000 |
| BSI: feeling alone; even when with other people_past week? | ordered | 'Not at all' to 'A little' | 896 | 0.033 | 0.006 | 0.061 | 0.0496 | 1.0000 |
| BSI: feeling impeded when doing all kinds of things_past week? | ordered | 'Not at all' to 'A little' | 892 | 0.032 | 0.007 | 0.057 | 0.0445 | 1.0000 |
| BSI: nausea or an upset stomach_past week? | ordered | 'Not at all' to 'A little' | 894 | 0.031 | 0.007 | 0.054 | 0.0381 | 1.0000 |
| BSI: feeling that others are watching you_past week? | ordered | 'Not at all' to 'A little' | 896 | 0.042 | 0.009 | 0.075 | 0.0454 | 1.0000 |
| BSI: difficulty in making decisions_past week? | ordered | 'Not at all' to 'A little' | 900 | 0.048 | 0.025 | 0.071 | 0.0010 | 0.0161 |
| BSI: difficulty in concentrating_past week? | ordered | 'Not at all' to 'A little' | 897 | 0.03 | 0.008 | 0.052 | 0.0365 | 1.0000 |
| BSI: feeling physically weak_past week? | ordered | 'Not at all' to 'A little' | 899 | 0.027 | 0.005 | 0.049 | 0.0479 | 1.0000 |
| BSI: feeling tense_past week? | ordered | 'Not at all' to 'A little' | 897 | 0.038 | 0.016 | 0.061 | 0.0086 | 0.3265 |
| BSI: thinking about death or dying_past week? | ordered | 'Not at all' to 'A little' | 895 | 0.042 | 0.009 | 0.075 | 0.0461 | 1.0000 |
| BSI: feeling uncomfortable in crowds_past week? | ordered | 'Not at all' to 'A little' | 897 | 0.064 | 0.025 | 0.102 | 0.0097 | 0.3869 |
| BSI: feeling uncomfortable in crowds_past week? | ordered | 'Not at all' to 'A little' | 895 | 0.043 | 0.014 | 0.071 | 0.0194 | 1.0000 |
| feeling so restless that you cannot sit still_past week? | ordered | 'Not at all' to 'A little' | 897 | 0.047 | 0.018 | 0.077 | 0.0119 | 0.4980 |
| Dutch understanding | ordered | 'Not at all' to 'Very good' | 766 | -0.108 | -0.193 | -0.022 | 0.0469 | 1.0000 |
| Dutch writing | ordered | 'Not at all' to 'Very good' | 762 | -0.082 | -0.147 | -0.016 | 0.0478 | 1.0000 |
| Have you ever had a period in which you were anxious or worried | unordered | 'Do not know' to 'No' | 888 | -0.128 | -0.228 | -0.028 | 0.0439 | 1.0000 |
| Have you ever had a period in which you felt down or depressed | unordered | 'Do not know' to 'No' | 889 | -0.151 | -0.266 | -0.035 | 0.0408 | 1.0000 |
| Regular purchase of new clothes_possesion | unordered | 'No' to 'Yes' | 925 | 0.054 | 0.016 | 0.092 | 0.0260 | 1.0000 |
| Car (or lease car)_possession | unordered | 'No' to 'Yes' | 925 | 0.059 | 0.012 | 0.106 | 0.0478 | 1.0000 |
| Having friends or family round to eat 1 or more times a month | unordered | 'No' to 'Yes' | 929 | 0.057 | 0.016 | 0.099 | 0.0294 | 1.0000 |
| Holiday away from home for 1 week or more each year | unordered | 'No' to 'Yes' | 923 | 0.085 | 0.035 | 0.134 | 0.0080 | 0.2863 |
| Prenatal supplementation of folic acid | unordered | 'no' to 'start 1st 10 weeks' | 791 | -0.06 | -0.109 | -0.012 | 0.0471 | 1.0000 |
| Prenatal supplementation of folic acid | unordered | 'no' to 'start periconceptional' | 791 | -0.076 | -0.124 | -0.028 | 0.0146 | 0.7164 |
| Do you feel part of the Dutch culture | ordered | 'Not at all' to 'Mostly' | 881 | -0.085 | -0.133 | -0.036 | 0.0077 | 0.2409 |
| Do you spend free time outside the home | ordered | 'Never' to 'Often' | 861 | -0.053 | -0.096 | -0.01 | 0.0479 | 1.0000 |
| Has the father tested positive for HIV/AIDS | unordered | 'Do not know' to 'No' | 892 | -0.091 | -0.157 | -0.025 | 0.0320 | 1.0000 |
| Has the father tested positive for HIV/AIDS | unordered | 'Do not know' to 'Yes' | 892 | -0.111 | -0.179 | -0.042 | 0.0120 | 0.5278 |
| Do you spend leisure time with Dutch people | ordered | 'None' to 'Several' | 853 | -0.086 | -0.133 | -0.039 | 0.0049 | 0.1173 |
| Taking part in traditional Dutch celebrations/festivals | ordered | 'Never' to 'Every month' | 856 | -0.058 | -0.106 | -0.011 | 0.0496 | 1.0000 |
| Threatened or attacked due to ethnicity | ordered | 'Disagree' to 'Slightly agree' | 709 | 0.074 | 0.017 | 0.13 | 0.0400 | 1.0000 |
| I sometimes feel really useless | ordered | 'Disagree' to 'Slightly agree' | 877 | 0.038 | 0.013 | 0.062 | 0.0178 | 0.9093 |
| I wish I could feel more respect for myself | ordered | 'Disagree' to 'Slightly agree' | 871 | 0.042 | 0.017 | 0.068 | 0.0087 | 0.3378 |
| BSI: Global Severity Index | numerical |  | 888 | 0.106 | 0.053 | 0.16 | 0.0017 | 0.0374 |
| Education father | ordered | 'Primary education' to 'Higher education' | 814 | -0.058 | -0.094 | -0.022 | 0.0120 | 0.5228 |
| Did your family have a (lease) car? | unordered | 'No' to 'Yes' | 839 | -0.056 | -0.1 | -0.012 | 0.0454 | 1.0000 |
| Did you go on holiday with the family one week or more a year | unordered | 'No' to 'Yes' | 842 | -0.066 | -0.109 | -0.022 | 0.0194 | 1.0000 |
| Did you have leisure equipment such as sports equipment or bicycle | unordered | 'No' to 'Yes' | 842 | -0.087 | -0.147 | -0.027 | 0.0239 | 1.0000 |
| Height mother | numerical |  | 983 | -0.003 | -0.005 | -0.001 | 0.0365 | 1.0000 |
| BSI: Hostility score | numerical |  | 883 | 0.066 | 0.021 | 0.11 | 0.0218 | 1.0000 |
| EMBU: My father praised me | ordered | 'No never' to 'Yes always' | 827 | -0.094 | -0.137 | -0.051 | 0.0006 | 0.0072 |
| EMBU: My mother praised me | ordered | 'No never' to 'Yes always' | 851 | -0.064 | -0.109 | -0.02 | 0.0256 | 1.0000 |
| EMBU: I think my father tried to make my childhood interesting | ordered | 'No never' to 'Yes always' | 827 | -0.058 | -0.094 | -0.022 | 0.0128 | 0.6135 |
| EMBU: I think my mother tried to make my childhood interesting | ordered | 'No never' to 'Yes always' | 861 | -0.06 | -0.098 | -0.022 | 0.0146 | 0.7298 |
| EMBU: I had the feeling that my father tried to comfort me | ordered | 'No never' to 'Yes always' | 808 | -0.06 | -0.094 | -0.025 | 0.0077 | 0.2460 |
| Pregnancy hypertension_mother or sisters | unordered | 'Do not know' to 'Yes' | 878 | -0.082 | -0.138 | -0.026 | 0.0239 | 1.0000 |
| EMBU: My father treated me in such a way that I felt ashamed | ordered | 'No never' to 'Yes always' | 816 | 0.088 | 0.022 | 0.154 | 0.0365 | 1.0000 |
| People in my family encouraged me to achieve things | unordered | Don't know' to other | 883 | -0.042 | -0.074 | -0.01 | 0.0378 | 1.0000 |
| There was someone in our family who made me feel special | unordered | Don't know' to other | 884 | -0.049 | -0.083 | -0.014 | 0.0260 | 1.0000 |
| Someone in our family wanted me to achieve something | unordered | Don't know' to other | 888 | -0.054 | -0.085 | -0.023 | 0.0074 | 0.2224 |
| I felt that I was loved | unordered | Don't know' to other | 887 | -0.054 | -0.085 | -0.024 | 0.0062 | 0.1744 |
| People in our family felt a bond between each other | unordered | Don't know' to other | 869 | -0.046 | -0.077 | -0.014 | 0.0258 | 1.0000 |
| People in our family looked after each other | unordered | Don't know' to other | 885 | -0.066 | -0.096 | -0.035 | 0.0006 | 0.0078 |
| Someone in our family believed in me | unordered | Don't know' to other | 872 | -0.055 | -0.086 | -0.024 | 0.0059 | 0.1583 |
| Our family was a source of strength and support | unordered | Don't know' to other | 883 | -0.041 | -0.073 | -0.009 | 0.0444 | 1.0000 |
| Immigration status | ordered | 2nd generation' to 'Came to NL after age 15' | 978 | 0.073 | 0.028 | 0.119 | 0.0121 | 0.5564 |
| Netto income household | ordered | '< 1200' to '> 2000' | 919 | -0.113 | -0.154 | -0.072 | 1.5664e-05 | 3.1327e-05 |
| FAD: we avoid talking about our worries and problems_family now | ordered | 'Totally disagree' to 'Agree' | 869 | 0.075 | 0.038 | 0.112 | 0.0013 | 0.0235 |
| How good is your Dutch speaking | ordered | 'Not at all' to 'Very good' | 891 | -0.152 | -0.214 | -0.09 | 0.0001 | 0.0006 |
| How good is your Dutch reading | ordered | 'Not at all' to 'Very good' | 888 | -0.144 | -0.208 | -0.08 | 0.0004 | 0.0040 |
| How good is your Dutch writing | ordered | 'Not at all' to 'Very good' | 888 | -0.144 | -0.2 | -0.087 | 5.4301e-05 | 0.0002 |
| Mothers prefer their babies not to fidget and wriggle so much | ordered | 'Strongly disagree' to 'Moderately agree' | 867 | 0.084 | 0.033 | 0.136 | 0.0116 | 0.4771 |
| Mothers should not insist that children do what they were asked | ordered | 'Strongly disagree' to 'Moderately agree' | 728 | 0.065 | 0.016 | 0.114 | 0.0365 | 1.0000 |
| BSI: Obsessive-Compulsive score | numerical |  | 877 | 0.082 | 0.048 | 0.116 | 0.0001 | 0.0008 |
| BSI: Paranoid Ideation score | numerical |  | 886 | 0.071 | 0.031 | 0.111 | 0.0070 | 0.2018 |
| BSI: Positive Symptom Total | numerical |  | 823 | 0.004 | 0.002 | 0.006 | 0.0008 | 0.0114 |
| Rank child amongst sibling | numerical |  | 986 | -0.074 | -0.129 | -0.02 | 0.0340 | 1.0000 |
| LLDI sum score | numerical |  | 870 | 0.008 | 0.002 | 0.014 | 0.0272 | 1.0000 |

**Supplementary Table 3: Main results in the Dutch sample (discovery + replication combined), not corrected for GSI**

| **Variable** | **Type** | **Range/contrast** | **n** | **B** | **CIL** | **CIU** | **p FDR** | **p Bonf** |
| --- | --- | --- | --- | --- | --- | --- | --- | --- |
| Restricted by your health or the pregnancy_moderate exercise? | ordered | 'Not restricted' to 'Severely restricted' | 1899 | 0.048 | 0.02 | 0.075 | 0.0264 | 0.7126 |
| Take account of my wishes and preferences during the birth | ordered | 'Disagree' to 'Agree' | 1309 | -0.085 | -0.121 | -0.049 | 0.0015 | 0.0048 |
| Have you suffered from vaginal blood loss_past 2 months? | ordered | 'Never' to 'Few days a week' | 1887 | 0.108 | 0.053 | 0.163 | 0.0093 | 0.1314 |
| In general_how would you describe your health | ordered | 'Poor' to 'Very good' | 1825 | -0.055 | -0.082 | -0.029 | 0.0036 | 0.0360 |
| Moderate exercise_restricted by health or pregnancy now | ordered | 'Not restricted' to 'Severely restricted' | 1825 | 0.059 | 0.032 | 0.087 | 0.0030 | 0.0248 |
| Nervous_how often_past month | ordered | 'Never' to 'Often' | 1831 | 0.047 | 0.019 | 0.075 | 0.0296 | 0.8736 |
| How often did you feel down and depressed_past month | ordered | 'Never' to 'Often' | 1706 | 0.052 | 0.023 | 0.081 | 0.0180 | 0.4146 |
| Vaginal blood loss_past 3 months | ordered | 'Never' to 'Few days a week' | 1713 | 0.059 | 0.025 | 0.093 | 0.0243 | 0.6243 |
| difficulty in remembering things_past week? | ordered | 'Not at all' to 'A little' | 1913 | 0.028 | 0.013 | 0.042 | 0.0149 | 0.2749 |
| Having friends or family round to eat 1 or more times a month | unordered | 'No' to 'Yes' | 1826 | 0.059 | 0.028 | 0.089 | 0.0115 | 0.1848 |
| Feel that Dutch have something against me_agreement | ordered | 'Disagree' to 'Slightly agree' | 1283 | 0.098 | 0.039 | 0.156 | 0.0345 | 1.0000 |
| Threatened or attacked due to ethnicity_agreement | ordered | 'Disagree' to 'Slightly agree' | 1290 | 0.082 | 0.034 | 0.13 | 0.0296 | 0.8869 |
| Concentration C203n9 as percentage of maternal total fatty acids | numerical |  | 1638 | -0.285 | -0.459 | -0.11 | 0.0422 | 1.0000 |
| Did your family invite guests for diner? | unordered | 'No' to 'Yes' | 1790 | -0.04 | -0.063 | -0.017 | 0.0243 | 0.6323 |
| Did your parents go out once every 14 days? | unordered | 'No' to 'Yes' | 1784 | -0.045 | -0.066 | -0.024 | 0.0030 | 0.0228 |
| EMBU: My father praised me | ordered | 'No never' to 'Yes always' | 1801 | -0.056 | -0.086 | -0.025 | 0.0174 | 0.3645 |
| EMBU: My mother praised me | ordered | 'No never' to 'Yes always' | 1842 | -0.056 | -0.088 | -0.024 | 0.0236 | 0.5657 |
| EMBU: I think my father tried to make my childhood interesting | ordered | 'No never' to 'Yes always' | 1794 | -0.046 | -0.07 | -0.021 | 0.0149 | 0.2714 |
| EMBU: I think my mother tried to make my childhood interesting | ordered | 'No never' to 'Yes always' | 1850 | -0.056 | -0.082 | -0.029 | 0.0038 | 0.0421 |
| EMBU: I had the feeling that my father tried to comfort me | ordered | 'No never' to 'Yes always' | 1767 | -0.04 | -0.064 | -0.017 | 0.0280 | 0.7847 |
| EMBU: I had the feeling that my mother tried to comfort me | ordered | 'No never' to 'Yes always' | 1823 | -0.048 | -0.074 | -0.021 | 0.0180 | 0.4066 |
| EMBU: my father showed me with words that he loved me | ordered | 'No never' to 'Yes always' | 1801 | -0.051 | -0.076 | -0.026 | 0.0047 | 0.0561 |
| EMBU: my mother showed me with words that he loved me | ordered | 'No never' to 'Yes always' | 1850 | -0.058 | -0.085 | -0.032 | 0.0026 | 0.0157 |
| I could discuss my problems with someone in my family | unordered | Don't know' to other | 1867 | -0.043 | -0.065 | -0.021 | 0.0093 | 0.1388 |
| People in my family encouraged me to achieve things | unordered | Don't know' to other | 1865 | -0.036 | -0.057 | -0.014 | 0.0374 | 1.0000 |
| I knew that there was someone who cared for me | unordered | Don't know' to other | 1868 | -0.04 | -0.061 | -0.018 | 0.0149 | 0.2834 |
| Someone in our family wanted me to achieve something | unordered | Don't know' to other | 1870 | -0.044 | -0.065 | -0.024 | 0.0030 | 0.0268 |
| I felt that I was loved | unordered | Don't know' to other | 1866 | -0.033 | -0.053 | -0.012 | 0.0456 | 1.0000 |
| People in our family felt a bond between each other | unordered | Don't know' to other | 1840 | -0.041 | -0.062 | -0.02 | 0.0093 | 0.1343 |
| People in our family looked after each other | unordered | Don't know' to other | 1871 | -0.046 | -0.067 | -0.026 | 0.0015 | 0.0058 |
| Someone in our family believed in me | unordered | Don't know' to other | 1867 | -0.046 | -0.066 | -0.026 | 0.0018 | 0.0088 |
| Our family was a source of strength and support | unordered | Don't know' to other | 1867 | -0.053 | -0.075 | -0.031 | 0.0008 | 0.0016 |
| It is frustrating to have to look after a newborn baby | ordered | 'Strongly disagree' to 'Moderately agree' | 1868 | 0.056 | 0.025 | 0.086 | 0.0172 | 0.3447 |
| Child should stop bottle or breastfeeding as soon as possible | ordered | 'Strongly disagree' to 'Moderately agree' | 1868 | 0.098 | 0.037 | 0.159 | 0.0458 | 1.0000 |
| Once mothers have decided on rules children have to obey | ordered | 'Strongly disagree' to 'Moderately agree' | 1562 | 0.037 | 0.014 | 0.06 | 0.0495 | 1.0000 |
| BSI: Obsessive-Compulsive score | numerical |  | 1902 | 0.066 | 0.025 | 0.106 | 0.0456 | 1.0000 |

**Supplementary Table 4: Main results in replication group, corrected for GSI**

| **Variable** | **Type** | **Range/contrast** | **n** | **B** | **CIL** | **CIU** | **p FDR** | **p Bonf** |
| --- | --- | --- | --- | --- | --- | --- | --- | --- |
| Have you felt calm and contented_past month? | ordered | 'Never' to 'Often' | 943 | -0.107 | -0.167 | -0.046 | 0.0088 | 0.0882 |
| Have you felt happy_past month? | ordered | 'Never' to 'Often' | 942 | -0.096 | -0.164 | -0.028 | 0.0405 | 0.8915 |
| In general_how would you describe your health | ordered | 'Poor' to 'Very good' | 928 | -0.051 | -0.089 | -0.014 | 0.0480 | 1.0000 |
| Nervous_how often_past month | ordered | 'Never' to 'Often' | 934 | 0.084 | 0.044 | 0.123 | 0.0011 | 0.0056 |
| Energetic_how often_past month | ordered | 'Never' to 'Often' | 928 | -0.07 | -0.108 | -0.031 | 0.0079 | 0.0712 |
| Physical or emotional problem hinder your activities_past month | ordered | 'Never' to 'Often' | 931 | 0.062 | 0.026 | 0.097 | 0.0106 | 0.1169 |
| Holiday away from home for 1 week or more each year | unordered | 'No' to 'Yes' | 923 | 0.079 | 0.029 | 0.128 | 0.0207 | 0.2897 |
| Prenatal supplementation of folic acid | unordered | 'no' to 'start periconceptional' | 791 | -0.07 | -0.118 | -0.022 | 0.0343 | 0.7199 |
| Feel part of the Dutch culture_agreement | ordered | 'Not at all' to 'Mostly' | 881 | -0.081 | -0.129 | -0.032 | 0.0161 | 0.1929 |
| Do you spend leisure time with Dutch people | ordered | 'None' to 'Several' | 853 | -0.074 | -0.121 | -0.027 | 0.0240 | 0.3593 |
| Education father | ordered | 'Primary education' to 'Higher education' | 814 | -0.054 | -0.09 | -0.019 | 0.0262 | 0.4709 |
| My father praised me | ordered | 'No never' to 'Yes always' | 827 | -0.086 | -0.13 | -0.043 | 0.0021 | 0.0155 |
| I had the feeling that my father tried to comfort me | ordered | 'No never' to 'Yes always' | 808 | -0.048 | -0.083 | -0.013 | 0.0499 | 1.0000 |
| Someone in our family wanted me to achieve something | unordered | Don't know' to other | 888 | -0.05 | -0.081 | -0.019 | 0.0190 | 0.2474 |
| I felt that I was loved | unordered | 'Don't know' to other | 887 | -0.045 | -0.075 | -0.014 | 0.0343 | 0.6976 |
| People in our family looked after each other | unordered | 'Don't know' to other | 885 | -0.06 | -0.09 | -0.03 | 0.0021 | 0.0164 |
| Someone in our family believed in me | unordered | 'Don't know' to other | 872 | -0.047 | -0.078 | -0.016 | 0.0261 | 0.4438 |
| Immigration status | ordered | 2nd generation' to 'Came to NL after age 15' | 978 | 0.068 | 0.022 | 0.113 | 0.0299 | 0.5686 |
| Netto income household | ordered | '< 1200' to '> 2000' | 919 | -0.104 | -0.145 | -0.063 | 7.7632e-05 | 0.0002 |
| How good is your Dutch speaking | ordered | 'Not at all' to 'Very good' | 891 | -0.144 | -0.205 | -0.082 | 0.0002 | 0.0009 |
| How good is your Dutch reading | ordered | 'Not at all' to 'Very good' | 888 | -0.133 | -0.197 | -0.069 | 0.0013 | 0.0076 |
| How good is your Dutch writing | ordered | 'Not at all' to 'Very good' | 888 | -0.135 | -0.191 | -0.079 | 0.0001 | 0.0004 |
| Mothers prefer their babies not to fidget and wriggle so much | ordered | 'Strongly disagree' to 'Moderately agree' | 867 | 0.079 | 0.028 | 0.13 | 0.0261 | 0.4182 |
